# Potential roles of the sirtuins in promoting longevity for larger *Argopecten* scallops

**DOI:** 10.1007/s42995-024-00269-3

**Published:** 2025-03-04

**Authors:** Yang Zhao, Junhao Ning, Yuan Wang, Guilong Liu, Xin Xu, Chunde Wang, Xia Lu

**Affiliations:** 1 Research and Development Center for Efficient Utilization of Coastal Bioresources, Yantai Institute of Coastal Zone Research, Chinese Academy of Sciences, Yantai, 264003 China; 2 School of Ocean, Yantai University, Yantai, 264005 China; 3 University of Chinese Academy of Sciences, Beijing, 100049 China; 4 College of Marine Science and Engineering, Qingdao Agricultural University, Qingdao, 266109 China; 5 Yantai Spring-Sea AquaSeed, Ltd., Yantai, 264006 China

**Keywords:** Scallops, *SIRTs*, Genetic mutation, Evolution, Nutrient restriction, Longevity

## Abstract

Annual bay scallops are commercially significant bivalve species for fisheries and aquaculture, but their small size and severe inbreeding depression impede the development of their industry. Some interspecific hybrids of bay scallops and peruvian scallops show longer lifespans and significantly greater sizes, which may result from the longevity genes in the latter (7–10 years). Sirtuins (*SIRTs*) play pivotal roles in the genetic control of aging in various model species and human beings. However, the role of *SIRTs* in longevity has not been systematically studied in aquatic animals. In this study, different gene numbers, sequences, structures and tandem duplications of *SIRTs* were first identified between the two scallops through genome-wide analysis. Cloning and characteristics of the *SIRT1* and *SIRT6* ORFs revealed dramatic variations in amino acids between the two scallops, which may cause intrinsic differences in function for longevity regulation. In particular, the amino acid variations in the N-terminus may auto-regulate conformations, causing intrinsic differences in catalytic activity for longevity regulation. The robust expression of *SIRT1* and *SIRT6-2* in peruvian scallops suggested they may exert a role in extending the lifespan. Nutrient restriction (NR) could promote lifespan in terrestrial model organisms, and the *SIRTs* and their related genes responded to NR for longevity in scallops; peruvian scallops showed a higher ability of autophagy. This study provides potential biomarkers for breeding long-lived larger scallop hybrids for the sustainability of aquaculture. Moreover, the genetic variation during evolution in the two scallops provides a foundation for further research on the longevity function of the *SIRTs*.

**Supplementary Information:**

The online version contains supplementary material available at 10.1007/s42995-024-00269-3.

## Introduction

The bay scallop (*Argopecten irradians*), which is naturally distributed along the Atlantic coast, has been a commercially important bivalve species for fishery and aquaculture in China since it was introduced in the late 1980s (Zhang et al. 2007). However, the small size and severe inbreeding depression hamper the sustainable development of aquaculture (Zheng et al. 2007, 2012). Peruvian scallops (*A. purpuratus*), a fast-growing bivalve, were introduced into China in 2007 and successfully hybridized with bay scallops to breed long-lived and larger scallops (Wang et al. 2017). Despite being taxonomically closely related bivalves, peruvian scallops and bay scallops exhibited distinction in terms of lifespan because of long-term adaption to varying natural environments over extended evolutionary periods (Chen et al. 2020b; Disalvo et al. 1984). The bay scallop has a relatively short-lived life span of no more than two years (Taylor and Capuzzo 1983), whereas the peruvian scallop can continue to grow after reaching sexual maturity in the first year with a lifespan of approximately 7–10 years (Waller 1969; Wang et al. 2011). Some of their interspecific hybrids may survive for 2 or even 3 years, and their weight is approximately 180% and 270% greater than that of bay scallops, respectively (unpublished data). Such huge advantages of these hybrids may be attributed to the longevity genes of peruvian scallops. Although there are many and diverse species with different lifespans in the ocean, research on the longevity of marine organisms are scarce. Especially, very few studies have been conducted on marine bivalves, although their simple organizational hierarchies and ontogeny make them well suited for such studies (Abele et al. 2009; Wang et al. 2022). Aquatic animals, especially marine bivalves with different lifestyles and diverse lifespans, such as *Argopecten* scallops, may be alternative novel model organisms for examining aging and longevity (Abele et al. 2009; Estabrooks 2007; Lian et al. 2019). In this regard, it will be of great significance to explore the genetic basis related to aging and lifespan in scallops during natural evolution to select long-lived and larger hybrids.

Sirtuins (*SIRTs*), also referred to as silent information regulator 2 (*SIR*2), are class III histone deacetylases that participate in various biological progresses (Michan and Sinclair 2007; Sinha 2019), such as insulin regulation, cellular senescence, telomere maintenance and lifespan control (Hirschey 2011; Imai and Armstrong 2000; Nogueiras et al. 2012). In the 1990s, *SIR*2 was initially discovered in the budding yeast, *Saccharomyces cerevisiae*, and its ability to extend the yeast lifespan was subsequently confirmed (Guarente and Kenyon 2000; Rine et al. 1979). Over the years, *SIRTs* have been progressively discovered, and their roles in longevity regulation identified (Nandave et al. 2023; Sulaiman et al. 2010). Recently, sirtuin gene homologues played important roles in the immune response of shrimp (Nie et al. 2020). Additionally, homologous genes of *SIRT6* have been identified from several teleost species, such as zebrafish (*Danio rerio*), turquoise killifish (*Nothobranchius furzeri*) and blunt snout bream (*Megalobrama amblycephala*) (Wu et al. 2024). These proteins exhibit evolutionary antiquity and possess a highly conserved structure throughout a wide variety of organisms (Brachmann et al. 2018; Frye 2000). Typically, there exist seven *SIRT* homologs (*SIRT1-7*), and each endows with distinctive functions, structural attributes and subcellular localization within mammalian cells. *SIRT1*, *6*, and *7* predominantly localize to the nucleus, whereas *SIRT3*, *4*, and *5* are primarily mitochondrial, and *SIRT2* is cytoplasmic (Finkel et al. 2009; Sinha et al. 2019).

The positive effects of *SIRTs* on longevity, especially *SIRT1* and *SIRT6*, are increasingly being emphasized in various model species via genetic methods. For example, several studies have generated *SIRT1*/*SIRT6* knockout mice and reported that the lifespan of these animals would be shortened, although the severity of the phenotype appears to be correlated with the genetic background (Cheng et al. 2003; Mostoslavsky et al. 2006; Peshti et al. 2017; Simon et al. 2019; Xiao et al. 2010). Overexpression of *SIRT6* has been shown to extend mammalian lifespan (Roichman et al. 2017). Nutrition restriction (NR), a reduction in food intake without malnutrition, is a promising way to extend both life- and health span in various species from yeast to mammals (Hursting et al. 2003). Sirtuins are important candidates for proteins that are sensitive to NR. In wild-type yeast, *Sir2* is required for NR to promote longevity (Kenyon 2010). Overexpressing *SIRT1* in mice exhibited similarly longer lifespans than control wild-type mice, but demonstrated enhanced metabolic parameters resembling those associated with NR (Bordone et al. 2007). Among the seven mammalian SIRT proteins, both SIRT1 and SIRT6 protein levels increased upon NR and anti-aging in various mouse tissues and human cell lines (Cohen et al. 2004; Kanfi et al. 2008a). For example, *SIRT1* deacetylated and up-regulated activities of several anti-aging modulators, including *FoxO*, *CAT* and *SOD* under various NR conditions, leading to favorable metabolic outcomes (Guarente 2013; Zia et al. 2021). *SIRT6* level was observed to rise in rats subjected to a nutrient-restricted diet (Kanfi et al. 2008b, 2012). In our previous research, we have determined the critical roles of the IIS signaling pathway and Ras/Raf/MEK/ERK cascade on longevity, particularly the different response patterns of *FoxO*, *PTEN*, *MEK1* and *IGF*/*IGF1R* genes in these two scallops under NR (Wang et al. 2022, 2023; Xu et al. 2022; Yuan et al. 2022).

The two *Argopecten* scallops offered a unique opportunity for investigating how key genetic factors contributed to distinct lifespans in such closely related species. So far, systematic research and analysis of the *SIRT1* and *SIRT6* subfamilies on longevity in aquatic animals have been limited. In this study, the *SIRT* gene family was investigated in bay scallops and peruvian scallops with their chromosome-level genomes. To elucidate the functions of the *SIRT1* and *SIRT6* subfamilies in longevity, a single copy of *SIRT1* and four copies of *SIRT6* were cloned and characterized, and analyzed for their expression in different tissues at 12 months of age when the bay scallops were near the end of life. Furthermore, the transcriptome expression of the *SIRT* gene family and its related target genes response to NR was detected in these two scallops*.* The findings hold substantial implications for elucidating the roles of the *SIRTs* in longevity in aquatic animals, ultimately conferring benefit to selecting larger hybrids as biomarkers for aquaculture practices.

## Materials and methods

### Animals

For this study, peruvian scallops and bay scallops were cultivated in the open sea in the Yang Ma Island area of Yantai, Shandong Province, China. The scallops were brought into the hatchery of Yantai Spring-Sea AquaSeed Co., Ltd. located in Lai Zhou, Yantai, Shandong Province, China in early spring and conditioned to mature. After approximately nine months of cultivation, the scallops were used for NR. The detailed cultivation and NR methods used were depicted in our previous work (Wang et al. 2022).

### Identification of the SIRT gene family in two scallop species

To acquire *SIRT* orthologs in *Argopecten* scallops, all members of the *SIRT* gene family, were identified through a screening process in the bay scallop genome (chromosome level) and peruvian scallop genome (haplotype-resolved chromosome level) (unpublished data) using the BLASTp program (version: blast-2.11.0 +). Simultaneously, the Hidden Markov Model (HMM) profile corresponding to the NAD^+^ binding domain (PF02146) was retrieved from the Pfam protein family database (https://pfam-legacy.xfam.org/), and used to query two scallop genome databases based on the expected value (E value) of 1 × 10^–5^ of HMMER 3.0 (Finn et al. 2011) to search for *SIRT* genes. The molecular weight (MW), theoretical isoelectric point (pI), and total average hydrophilicity (GRAVY) were detected by the online software Prosite ExPASy server (http://web.expasy.org/protparam/) (Wilkins et al. 1999). Other characteristics, such as gene location, coding sequence length (bp) and number of amino acids (aa), were obtained from two scallop genome databases.

### Chromosomal localization and phylogenetic analysis of the SIRT gene family in two scallop species

The sequences of the identified AiSIRT (in bay scallops) and ApSIRT (in peruvian scallop) proteins, as well as 87 additional SIRT proteins from humans, mouse, *Caenorhabditis elegans*, *Danio rerio*, *Drosophila melanogaster*, *Mytilus edulis* and *Mizuhopecten yessoensis* that were obtained from the NCBI database (https://www.ncbi.nlm.nih.gov/) were used for phylogenetic analysis (Liu et al. 2022). The muscle method was employed to align multiple protein sequences. The phylogenetic tree was constructed with MEGA X software through the neighbor-joining method with 1000 bootstrap replicates (González-Fernández et al. 2024). Subsequently, the tree was visualized and beautified by iTOL (https://itol.embl.de/) (Letunic and Bork 2021). The chromosomal scaffold location for each verified *SIRT* gene was extracted from the GFF3 data of two scallop species and analyzed using TBtools software (version 1.120) (Chen et al. 2020a).

### Analysis of motifs and gene structures of SIRT proteins in two scallop species

The conserved motifs in two scallop species of the SIRT proteins were conducted by the Multiple Expectation Maximization for Motif Elicitation (MEME) software (https://meme-suite.org/) (Timothy et al. 2015). The positions of exons, introns and untranslated regions (UTRs) for each *SIRT* gene were sourced from the GFF3 genomes. The exon/intron structure of the *SIRT* genes, conserved motifs and structural domains of SIRT proteins were visualized using TBtools software. The name of each gene began with Latin abbreviation for species and the gene loci were renumbered based on the order of the 1–7 subgroups.

### Genomic distribution map and Ka/Ks calculations of SIRT1 and SIRT6

As *SIRT1* and *SIRT6* have been shown to be involved in regulating longevity in terrestrial model organisms, we focused mainly on these two genes in subsequent research. Utilizing the genome annotation file, gene duplication and synteny analysis of *SIRT1* and *SIRT6* were performed with TBtools software (Chen et al. 2020a). To test the effect of selection pressure on the *SIRT1* and *SIRT6* protein-coding genes, the Ka/Ks calculator in TBtools was used to calculate the synonymous (Ks) and nonsynonymous (Ka) substitution rates of each pair of homologous genes and the Ka/Ks ratios. The value Ka/Ks < 1 represents negative or purifying selection, Ka/Ks equal to 1 represents neutral selection, and Ka/Ks > 1 represents positive selection (Peng et al. 2021).

### Cloning, sequence analysis and phylogenetic study of SIRT1 and SIRT6 genes in two scallop species

Total RNA was separately extracted from adductor muscles of the two scallop species using Transzol (ET101-01-V2), and its quality was checked by 1% agarose gel electrophoresis and a Nanodrop 2000 spectrophotometer (NanoDrop Technologies, USA), respectively. The complementary DNA (cDNA) for gene cloning was synthesized from total RNA (1 μg) using the PrimeScript RT reagent kit (TaKaRa, Japan) following the manufacturer’s protocol.

To amplify the CDSs of the *SIRT1* and *SIRT6* genes, primers were designed using Primer 5.0 based on the CDSs of *SIRT* from the whole genomes of these two scallops (data unpublished) (Supplementary Table S1). PCR amplification was performed in a total volume of 50 μL, containing 20 ng of cDNA as a template, 2 μL of each primer, 25 μL of Premix Taq™ (TaKaRa, Japan), and 19 μL of ddH_2_O. All the samples were denatured for 5 min at 95 °C, followed by 35 cycles of 98 °C for 10 s, 52 °C for 15 s, and 72 °C for 2 min, with a final extension at 72 °C for 7 min. The PCR products were separated by electrophoresis on a 1.5% agarose gel and the target bands were purified using a D2500 Gel Extraction Kit (OMEGA, USA). The purified DNA was then ligated into the pTOPO-TA vector (Aidlab, Beijing, China) and sequenced. The purified DNA was subsequently ligated into the pEASY^®^-Blunt Zero Cloning Vector (TransGen Biotech) and sequenced by Sangon Biotech Co., Ltd. (Qingdao, China).

The open reading frames (ORFs) were identified by the Open Reading Frame Finder tool on NCBI (http://www.ncbi.nlm.nih.gov), and the ORFs were translated into amino acid sequences using DNAMAN 9.0 (Yu et al. 2020). The conserved domains were predicted by the Simple Modular Architecture Research Tool (https://www.smart.emblheidelbergde/). The amino acid sequences of SIRT1 and SIRT6 from 21 other species were selected from the NCBI database (http://www.ncbi.nlm.nih. gov). Among these, 5 species (*D. melanogaster*, *M.edulis*, *M. yessoensis*, *R.norvegicus*, and humans) were selected for alignment with the deduced amino acid sequences of SIRT1 and SIRT6 in the two scallop species by DNAMAN 9.0 software, and a neighbor-joining phylogenetic tree was created with 1000 replications bootstrap using MEGA X with all 21 sequences.

### Tertiary structure model analysis and subcellular localization of SIRT1 and SIRT6

SOPMA (http://npsa-pbil.ibcp.fr/cgi-bin/npsa_automat.pl?page=npsa_sopma.html) was used to predict the secondary structures of the SIRT1 and SIRT6 proteins. FASTA formatted amino acid sequences were submitted to the Phyre2 web portal through homology modeling for prediction with default parameters in building protein three-dimensional (3D) structures. The final structures were visualized using PyMOL software (The PyMOL Molecular Graphics System, Schrodinger, LLC) (https://pymol.org/edu/). Subcellular localization prediction for the *SIRT1* and *SIRT6* genes was carried out using both WoLF PSORT (https://wolfpsort.hgc.jp/) and Cell-PLoc2.0 (http: //www.csbio.sjtu.edu.cn/bioinf/Cell-PLoc-2/), and SIRT1 and SIRT6-2 were selected for validation of their subcellular localization in the two scallop species. SIRT1 and SIRT6-2 (without the stop codon: TGA) were fused into the N terminus of GFP to construct the pEGFP-N1-SIRT1 and pEGFP-N1-SIRT6-2 fusion expression vectors, respectively, in the two scallops. The vectors were introduced into the HEK293T cells, and then the transfected cells were imaged using laser confocal microscopy (FV300, Olympus).

### Expression of SIRT1 and SIRT6 in two scallop species

To examine the expression of *SIRT1* and *SIRT6* in the two scallops at the 12-month-old stage when bay scallops near the end of life, six bay scallops and six peruvian scallops were collected and transported to the laboratory. Adductor muscles, gills, gonads, hepatopancreases, and mantles were dissected from each animal and immediately frozen in liquid nitrogen and stored at −80 °C for subsequent analysis.

Total RNA was extracted separately from each tissue using the method described above. First-strand cDNA was synthesized with HiScript III RT SuperMix for qPCR (+ gDNA wiper) (R323) and Taq Pro Universal SYBR qPCR Master Mix (Vazyme Biotech, China) (Q712-02) was used for qRT-PCR according to the manufacturer’s guidelines. The qRT-PCR was conducted with the following reaction mixture: 5 µL of 2 × ChamQ Universal SYBR qPCR Master Mix (Vazyme, China), 3.6 µL of H_2_O, 0.2 µL of each primer, and 1 µL of cDNA. The amplification program was set at 95 °C for 30 s; 40 cycles of 95 °C for 10 s, 60 °C for 30 s; 95 °C for 15 s, 60 °C for 60 s, and 95 °C for 15 s. Primers of *SIRT1* and *SIRT6* were designed by Primer 5.0 software and the list of primers utilized in this study was available in Supplementary Table S1. The results were calculated using the 2^−ΔΔCt^ method with the elongation factor (*EF1-α*) as the internal control.

### Expression of the SIRT gene family and its related genes under NR

To detect the differences in the expression of the *SIRT* gene family and its related genes in response to NR in two scallop species with distinct lifespans, RNA-seq was performed to evaluate their mRNA expression in the NR and control groups of the two scallops at Day 56, respectively. The hepatopancreas of three individuals from each group of the two scallops were dissected separately and immediately frozen in liquid nitrogen for subsequent RNA extraction as described above. The mRNA of each individual was subjected to library construction and sequencing according to the methods described in our previous study (Yu et al. 2023). Differentially expressed genes (DEGs) between treatment and control groups were identified using the DESeq R package (1.18.0). To control the false discovery rate, the resulting* P* values were adjusted by Benjamini and Hochberg’s approach, and genes with an adjusted *P* value < 0.05 and |log_2_ (fold change) |> 1.0 were assigned as DEGs. The *SIRT* gene family and its related genes that have been reported in previous studies of the model organisms and humans were screened out from the DEGs.

## Results

### Identification and characterization of SIRT genes in the two scallops

We extracted the *SIRT* genes from the genomes of bay scallops and peruvian scallops using the full-length protein sequences of 87 genes from humans, mouse, *C. elegans*, *D. rerio*, *D. melanogaster*, *M. edulis* and *M. yessoensis* as queries by the two BLASTp methods. As a result, the gene number, sequence and MW of the *SIRT* genes were different between the bay scallop and peruvian scallop (Supplementary Table S2). 15 *SIRT* genes were identified in bay scallops but 11 *SIRT* genes were identified in peruvian scallops. Among them, the *SIRT* genes were uniformly named as *AiSIRT* for bay scallops and *ApSIRT* for peruvian scallops. The CDS of these genes ranged from 798 bp to 2,382 bp, whereas their corresponding proteins ranged from 265 to 793 aa, and showed different physicochemical properties of the homologous genes between the two scallops (Supplementary Table S2**)**. *AiSIRT5-*25,150.1 exhibited the lowest MW of 28 kDa, whereas *AiSIRT1-*15,044.1 displayed the highest MW of 87 kDa among the *SIRT* genes. The pIs of the *SIRT* proteins varied from 4.36 to 9.94. All the SIRT proteins exhibited GRAVY values below zero, indicating that these proteins were hydrophilic.

### Chromosome distribution and phylogenetic analysis of SIRT genes in two scallop species

It was observed that most *SIRT* genes located on Chromosome 4 (Chr4), but the number and location of the genes on the homologous chromosomes were different between bay scallops and peruvian scallop, such as the Chr3, Chr4, and Chr6 (Supplementary Fig. S1). Furthermore, our analysis revealed the presence of four sets of tandem duplicated (TD) genes, with three sets attributed to bay scallops (Chr3, Chr4 and Chr6) and one set to peruvian scallops (Chr3). The *SIRT2* genes were two copies on peruvian scallop chromosomes (rna-Apu33416.1, rna-Apu3315.1) on the Chr3. However, there were three copies of *SIRT2* (rna-Air14084.1, rna-Air14083.2, rna-Air14083.3) on the Chr3 of the bay scallop.

**Fig. 1:**
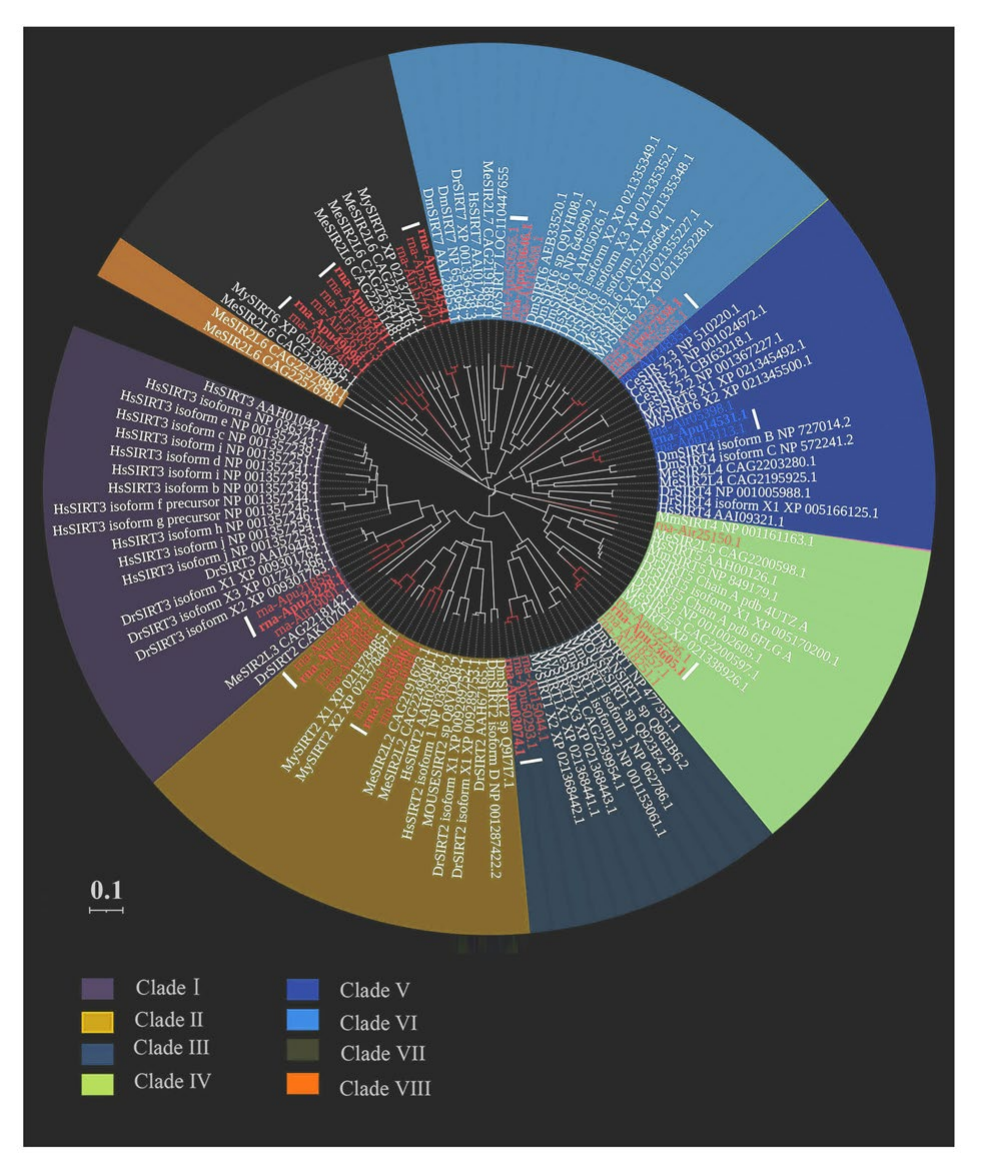
Phylogenetic tree of *SIRT* genes from two scallop species and 87 other species. Different colors represent different groups. The white lines indicate alleles on homologous chromosomes of peruvian scallops

A phylogenetic tree comprising 37 SIRT proteins (15 genes in bay scallops and 11 pairs in peruvian scallops) from the scallops and 87 proteins from other species was constructed*.* The *SIRT* gene family was divided into eight clades (I to VIII). Specifically, clade I encompassed two members, clade II contained five members, and clade III comprised two members. Furthermore, clades IV, V, VI, and VII accommodated four, three, four, and six members, respectively (Fig. 1). *AiSIRT5*-25,150.1 and *AiSIRT4*-24,835.1 were situated in clades IV and V, respectively, despite their absence within the assembled chromosomes.

### Motif identification and gene structures analysis of SIRT proteins

**Fig. 2:**
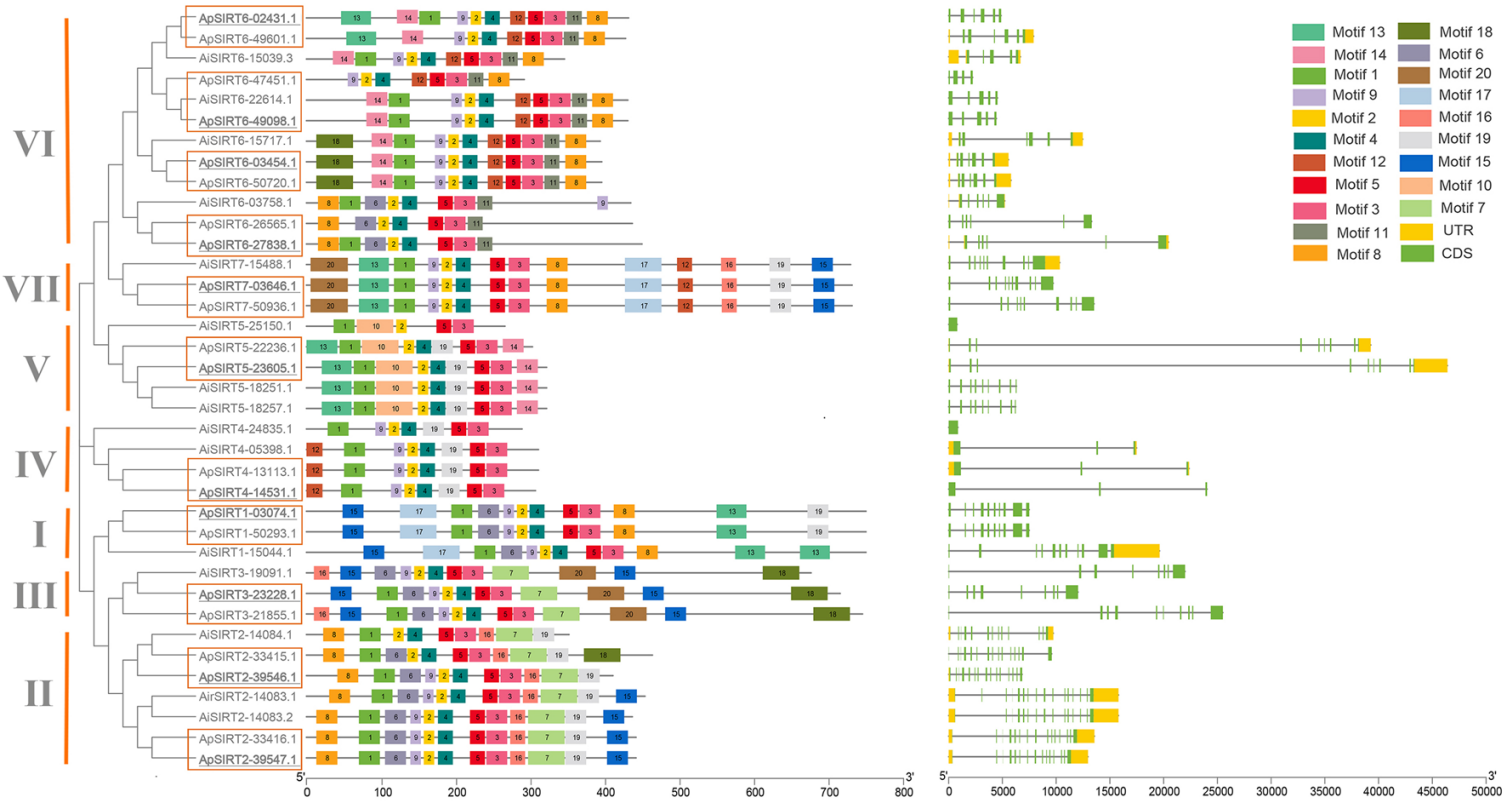
Phylogenetic tree and gene structures of *SIRT* genes in two scallop species. The *SIRT* structures of intron and exon and UTR were shown in gray line, green, and yellow boxes, respectively

A total of 20 potential motifs were identified in the *SIRT* gene family, as illustrated in Fig. 2. The number of motifs of the *SIRT* gene family ranged from 5 to 20. In general, *SIRT* genes within the same clusters displayed analogous motif arrangements, but there were some differences in some members between the two scallops. Compared to *ApSIRT1*, motif 19 was missing in *AiSIRT1*. The *AiSIRT2*-14,083.2 and *AiSIRT2*-14,083.1 had the same motifs with *ApSIRT2*-33,416.1*/ApSIRT2*-39,546.1, but *AiSIRT2*-14,084.1 lost motif 6 compared to *ApSIRT2*-39,546.1/*ApSIRT2*-33,415.1. *AiSIRT4*-24,835.1 and *AiSIRT5*-25,150.1 were absent motif 12 and motifs 4, 13, 14, and 19 in their corresponding clades, respectively. Remarkably, *AiSIRT6*-15,039.3 and *ApSIRT6*-47,451.1 were represented substantive changes in their respective branches. *SIRT3* and *SIRT7* exhibited high consistencies in their clades in the two scallops. Motifs 2, 3, and 5 were consistently present across all the SIRT proteins, underscoring the robust conservation of these domains within the *SIRT* genes of both scallop species.

Based on the gene structure information, the CDS-intron structure of each *SIRT* gene was depicted in Fig. 2**.** These genes exhibited a diverse range of CDS counts, from 1 to 16, and they varied between the two scallops. Importantly, the variability in CDS-intron structures among these *SIRT* genes underscores their potential diversity in biological functions. For example, *ApSIRT1* and *ApSIRT7* genes were not found in the UTR region as compared to *AiSIRT1* and *AiSIRT7*. All the copies of *AiSIRT5* genes lacked UTR region, but there were two UTR regions of *ApSIRT5*.

### Gene duplication, Ka/Ks calculations, and synteny analysis of SIRT gene family

**Fig. 3:**
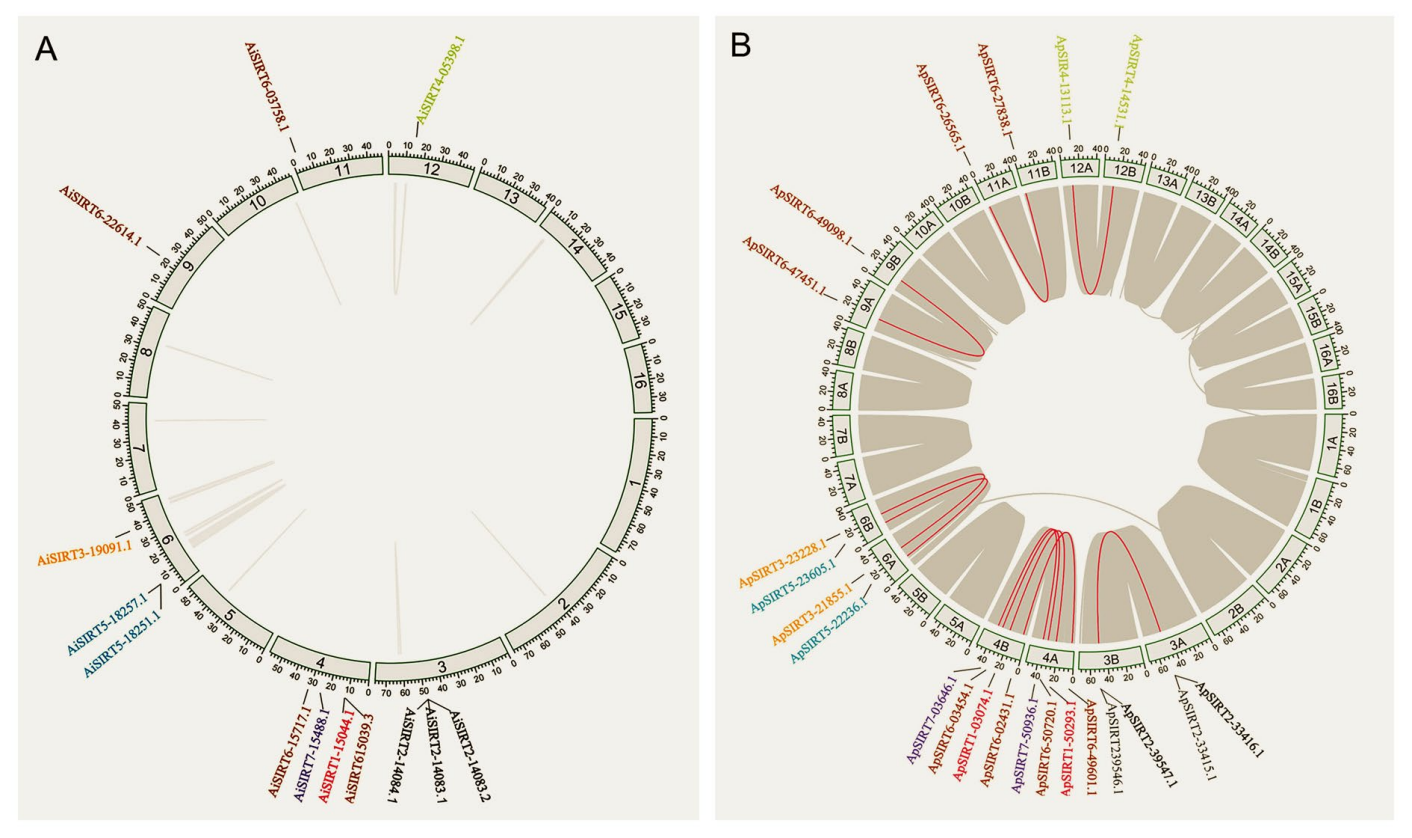
The distribution and duplication events of *SIRT1* and *SIRT6* in the bay scallop genome **A** and peruvian scallop genome **B** with two homologous chromosomes. The gray lines connect the relevant pairs of paralogous genes. The red lines connect the relevant pairs of *SIRT1-7* paralogous genes. The letters “A and B” in Figure B represent two homologous chromosomes of peruvian scallops

The *SIRT1* and *SIRT6* were single- and four copies in both two scallop species, respectively (Fig. 3). Notably, the Ka/Ks ratios for the *SIRT1* and *SIRT6* genes were computed to be < 1 (0.09–0.29), suggesting that purifying selection played a crucial role during evolution in these two scallops (Supplementary Table S3).

### Cloning and sequence analysis of AiSIRT1 and ApSIRT1

**Fig. 4:**
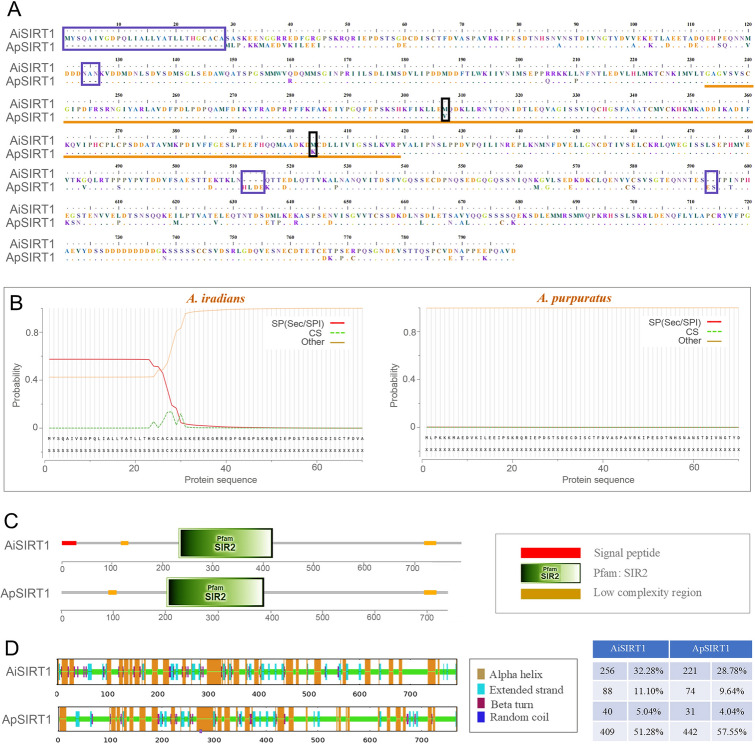
The sequence alignments **A**, the signal peptide **B**, the structure domains **C**, and the secondary structures of AiSIRT1 and ApSIRT1 **D**. The changes in the SIRT domain and the amino acids are marked by an orange line and a black box, respectively. The purple box represents the InDels

The ORFs of the *AiSIRT1* and *ApSIRT1* were 2,382 bp and 2,307 bp (accession nos. PP001051 and OR994705), encoding 793 and 768 amino acids, respectively. The MWs of the deduced proteins of AiSIRT1 and ApSIRT1 were 87.6 kDa and 85.2 kDa, and their PIs were 4.36 and 4.41, respectively. There were some variations in protein sequence between AiSIRT1 and ApSIRT1, containing an additional specific sequence (1–28 aa) located at the N-terminal domain in AiSIRT1, 63 synonymous SNPs and 61 nonsynonymous SNPs in the homologous region (Supplementary Fig. S2). As shown in Fig. 4A, there were four InDels between the nucleotide sequences of *ApSIRT1* and *AiSIRT1*, leading to 28 and 3 amino acids lacking in the protein ApSIRT1 N- terminus, 4 and 2 amino acids lacking in the protein AiSIRT1 C-terminus, respectively. Previous studies have indicated that the sequence divergent N- and C-terminal regions of the eukaryotic SIRT1 proteins may exert a particularly important role in their distinct substrate-binding properties, biological activities or both (Zhao et al. 2003). Therefore, the differences in the activities of ApSIRT1 and AiSIRT1 may well result from differences in the regions flanking the core domain.

SMART analysis demonstrated that both ApSIRT1 and AiSIRT1 belonged to the *SIRT* gene family, with a SIR domain positioned at amino acids 233–419. Apparently, the additional specific sequence at the N-terminus of AiSIRT1 contained 28 extra amino acids, which was predicted to constitute a signal peptide (Fig. 4B). In addition, there were two low complexity regions in both AiSIRT1 and ApSIRT1 (Fig. 4C). Prediction of the secondary structures of AiSIRT1 and ApSIRT1 revealed that differences existed between the two species (Fig. 4D).

### Cloning and sequence analysis of AiSIRT6 and ApSIRT6

The four copies cDNA sequences of *ApSIRT6* and *AiSIRT6* were verified via PCR and the NCBI database. Thus, they were separately named as *AiSIRT6-1*, *AiSIRT6-2*, *AiSIRT6-3*, and *AiSIRT6-4* in *A. irradians* and *ApSIRT6-1*, *ApSIRT6-2*, *ApSIRT6-3*, and *ApSIRT6-4* in *A. purpuratus*. As shown in Table 1, the four copies of the complete ORFs of *ApSIRT6* and *AiSIRT6* were 987 (*AiSIRT6-4*) bp to 1,350 (*ApSIRT6-3*) bp, encoding 328 (*AiSIRT6-4*) to 449 (*ApSIRT6-3*) amino acids in length, with putative MWs ranging from 36.8 kDa (*AiSIRT6-4*) to 50.3 (*ApSIRT6-3*) kDa. The pIs for eight proteins were 5.93 (*ApSIRT6-3*) to 8.53 (*AiSIRT6-4*).

**Table 1:** Characteristics of the *SIRT6* genes in two scallop species

Gene Name	Size of amino acid (aa)	Coding sequences Length (bp)	PI	MV (kDa)	Accession number
AiSIRT6-1	430	1293	6.17	49.1	PP001053
ApSIRT61	430	1293	6.03	49.3	PP001052
AiSIRT6-2	393	1182	8.09	44.0	PP024722
ApSIRT62	395	1188	8.11	44.3	PP001054
AiSIRT6-3	434	1350	6.07	48.3	PP024723
ApSIRT63	449	1305	5.93	50.3	PP024724
AiSIRT6-4	328	987	8.53	36.8	PP024726
ApSIRT64	431	1296	8.34	48.3	PP024725

There were some variations in sequence between the four copies of *AiSIRT6* and *ApSIRT6*, as summarized in Table 2. No Indel occurred in *SIRT6-1* (Supplementary Fig. S3) and one InDel occurred between the nucleotide sequences of *AiSIRT6-2* and *ApSIRT6-2*, resulting in the loss of S394 and Q395 in *AiSIRT6-2* (Supplementary Fig. S4). There were dramatic InDel changes in sequence between *AiSIRT6-3* and *ApSIRT6-3* in the C-terminal region, including two InDels in *ApSIRT6-3* and five in *AiSIRT6-3*, resulting in 21 different amino acids (Supplementary Fig. S5). In addition, *ApSIRT6-4* had an additional specific sequence (1–310 aa) located at the N-terminal domain, referred to as a large InDel variations (Supplementary Fig. S6).

**Table 2:** Variations of the *SIRT6* genes in two scallop species

Gene name		synonymous SNPs	nonsynonymous SNPs	Amino acids variations from SNPs	InDels
*AiSIRT6-1*	*ApSIRT6-1*	42	30	26	0
*AiSIRT6-2*	*AiSIRT6-2*	40	15	14	1
*AiSIRT6-3*	*AiSIRT6-3*	40	19	17	7
*AiSIRT6-4*	*ApSIRT6-4*	38	16	15	1

SMART analysis demonstrated that four copies of both ApSIRT6 and AiSIRT6 belonged to the *SIRT* gene family, with a SIR domain positioned at amino acids 118–393 aa, 120–288 aa, 52–268 aa, 158–396 aa for ApSIRT6-1 and AiSIRT6-1 (Supplementary Fig. S7A, B), ApSIRT6-2 and AiSIRT6-2 (Supplementary Fig. S8A, B), ApSIRT6-3 and AiSIRT6-3 (Supplementary Fig. S9A, B), and ApSIRT6-4 and AiSIRT6-4 (Supplementary Fig. S10A, B), respectively. There were several variations in the sequences between the four copies of ApSIRT6 and AiSIRT6, especially in the SIR domains (Supplementary Figs. S7A, 8A, 9A, 10A). In particular, the C-terminal regions of *AiSIRT6-3* and *ApSIRT6-3* were largely different. ApSIRT6-4 had an additional 310 aa insert from the N-terminal start site and H175 in ApSIRT6-4 was N175 in AiSIRT6-4. 13, 6, 4 and 8 amino acid variations were found in the SIR domain. There were some differences in the secondary structures of the SIRT6 proteins between the two scallops (Supplementary Figs. S7C, 8C, 9C, 10C).

### Amino acid alignment and phylogenetic analysis of SIRT1 and SIRT6 genes

The SIRT protein family is characterized by two amino acid sequence motifs: GAGISTS (L/A) GIPDFR and YTQNID (Brachmann et al. 2018). As depicted in Supplementary Figs. S11, 12, both the SIRT1 and SIRT6 proteins exhibited the essential motifs. Multiple alignments of SIRT1 protein sequences demonstrated that the two necessary motifs were well-conserved in all the species analyzed, and several additional amino acid motifs were presented among the SIRT1 family members, such as two nuclear localization signals (NLSs), a conserved SIR2 domain and one nuclear export signal (NES) (Supplementary Fig. S11). In common with *C. elegans* Sir2.1, ApSIRT1 and AiSIRT1 possessed a possible NLS and NES at the amino acid level, respectively. However, the NLS sequences of ApSIRT1 and AiSIRT1 were different. The four cysteine residues of the proposed metal-binding motif were highly conserved across all SIRT1 sequences (Supplementary Fig. S11). This conservation suggested the potential functional significance of these residues. Sequence alignment revealed a similarity of 72.77% between ApSIRT1 and AiSIRT1.

The sequence alignment indicated that the similarities of SIRT6-1, SIRT6-2, SIRT6-3 and SIRT6-4 between the two scallops were 93.95%, 95.95%, 90.93%, and 73.09%, respectively. The essential motifs of SIRT6 displayed low conservation and exhibited changes in two scallop species (Supplementary Fig. S12), implying that SIRT6 may have a distinct role in lifespan regulation. Additionally, the proposed metal-binding motif included four cysteine residues that were also present in various species. The PKRVKAK sequence located at the C-terminus of SIRT6 functions as an NLS crucial for accurate nuclear localization, which was different from SIRT1 where it was identified as NLS on the N-terminal extension.

The phylogenetic tree revealed the presence of three subgroups, identified as the *C. elegans* SIR superfamily, SIRT1 superfamily, and SIRT6 superfamily. These demonstrated a close relationship between *ApSIRT1* and *AiSIRT1* and next to *M. yessoensis*, followed by other vertebrates with extended lifespans. *ApSIRT6-1* and *AiSIRT6-1* were found to be closely related to *Mytilus coruscus*, followed by *ApSIRT6-2*, *AiSIRT6-2*, *ApSIRT6-4*, and *AiSIRT6-4* (Supplementary Fig. S13).

### 3D structure and subcellular localization analysis of SIRT1 and SIRT6 proteins in two scallop species

**Fig. 5:**
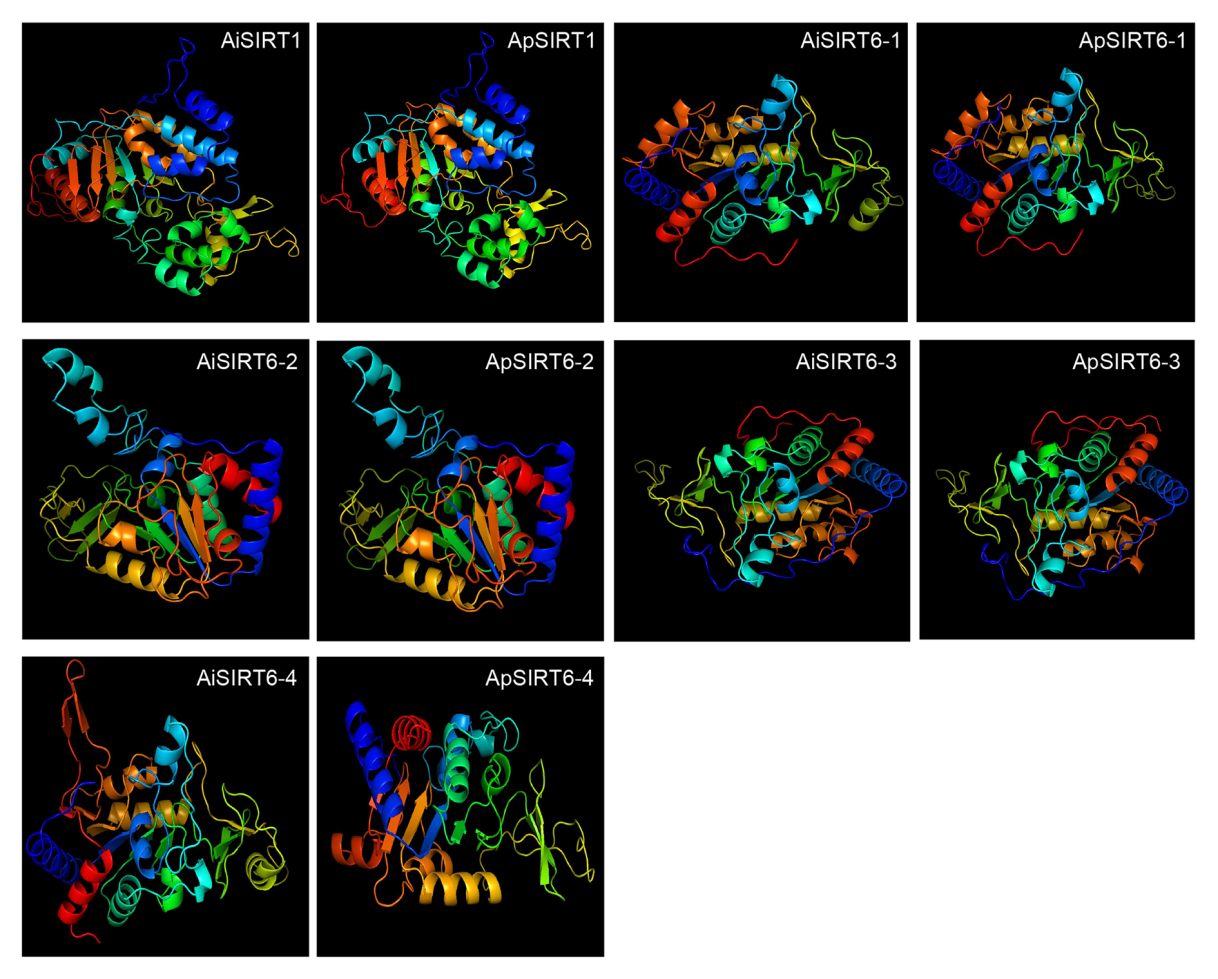
The tertiary structures of the SIRT1 and SIRT6 proteins were determined through PyMOL

Structural elucidation of proteins is vital for determining their specific functions. 3D homology modeling of ApSIRT1 and AiSIRT1 through Phyre2predicted tertiary structures highly similar to those of yeast Sir2 proteins (Zhao et al. 2003). The results showed that SIRT6 proteins exhibited similar structures to the same copy in two scallop species except for SIRT6-1 and SIRT6-4, whereas the structures appeared to be different between different copies of SIRT6 within species. These differed slightly in structures from ApSIRT1 and AiSIRT1 (Fig. 5). Collectively, the proteins from different species within the same subfamily exhibited similar structures, whereas those from different subfamilies within the same species exhibited considerable differences. The results revealed the structural diversity of the SIRT family in these two species.

**Fig. 6:**
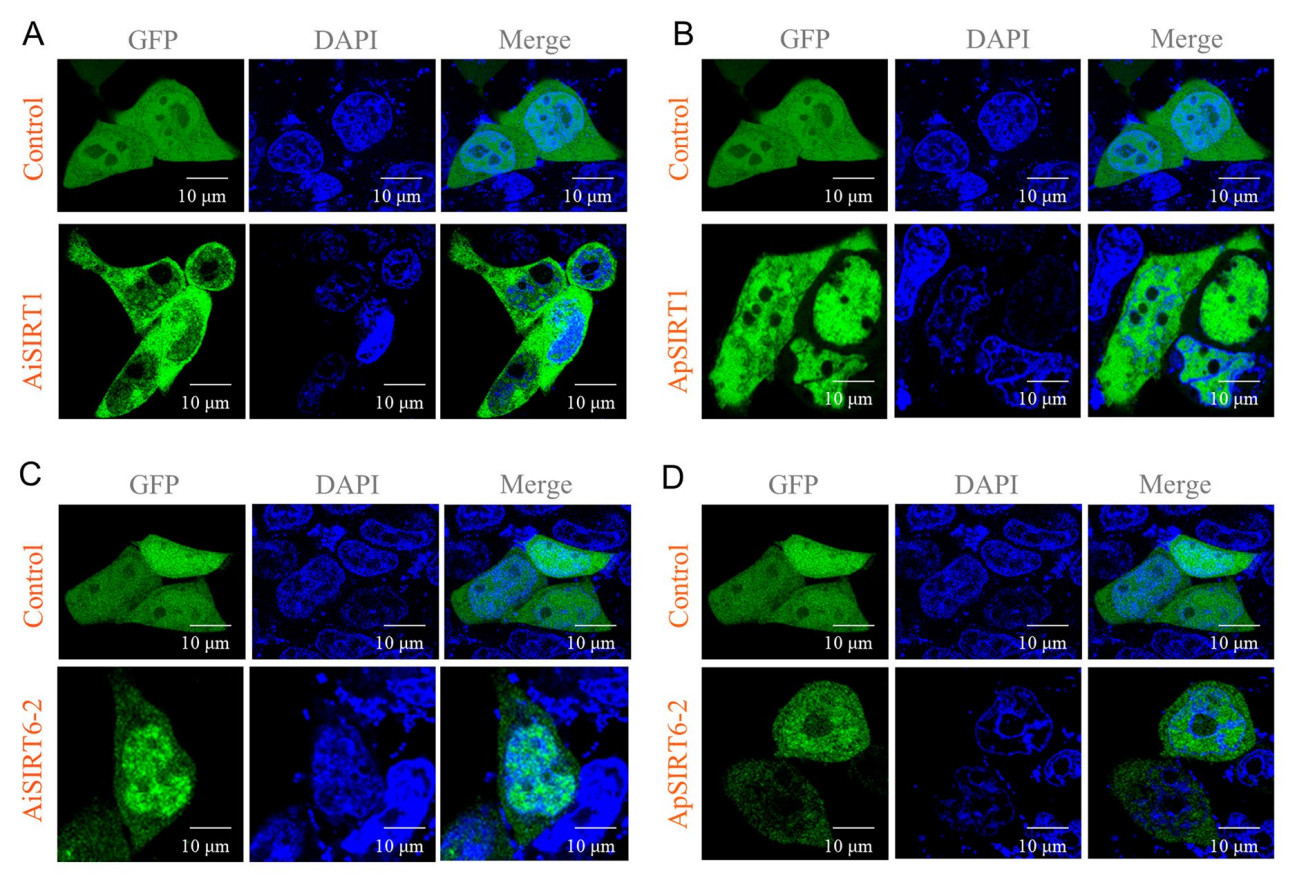
Subcellular localization of the SIRT1 and SIRT6-2 proteins in two scallop species. **A** Subcellular localization of AiSIRT1. **B** Subcellular localization of ApSIRT1. **C** Subcellular localization of AiSIRT6-2. **D** Subcellular localization of ApSIRT6-2. GFP signals show subcellular localization of selected proteins. Bars = 10 μm

Online prediction of subcellular localization indicated that the antiaging star members of the SIRT1 and SIRT6 proteins were situated within the nucleus and cytoplasm (Supplementary Table S4). Subsequently, we validated the subcellular localization of SIRT1 and SIRT6-2 in HEK 293 T cells. A green fluorescence signal was observed for AiSIRT1 (Fig. 6A), ApSIRT1 (Fig. 6B), AiSIRT6-2 (Fig. 6C), and ApSIRT6-2 (Fig. 6D), showing they located mainly in the cell nucleus.

### Expression patterns of SIRT1 and SIRT6 genes in different tissues in two scallops

**Fig. 7:**
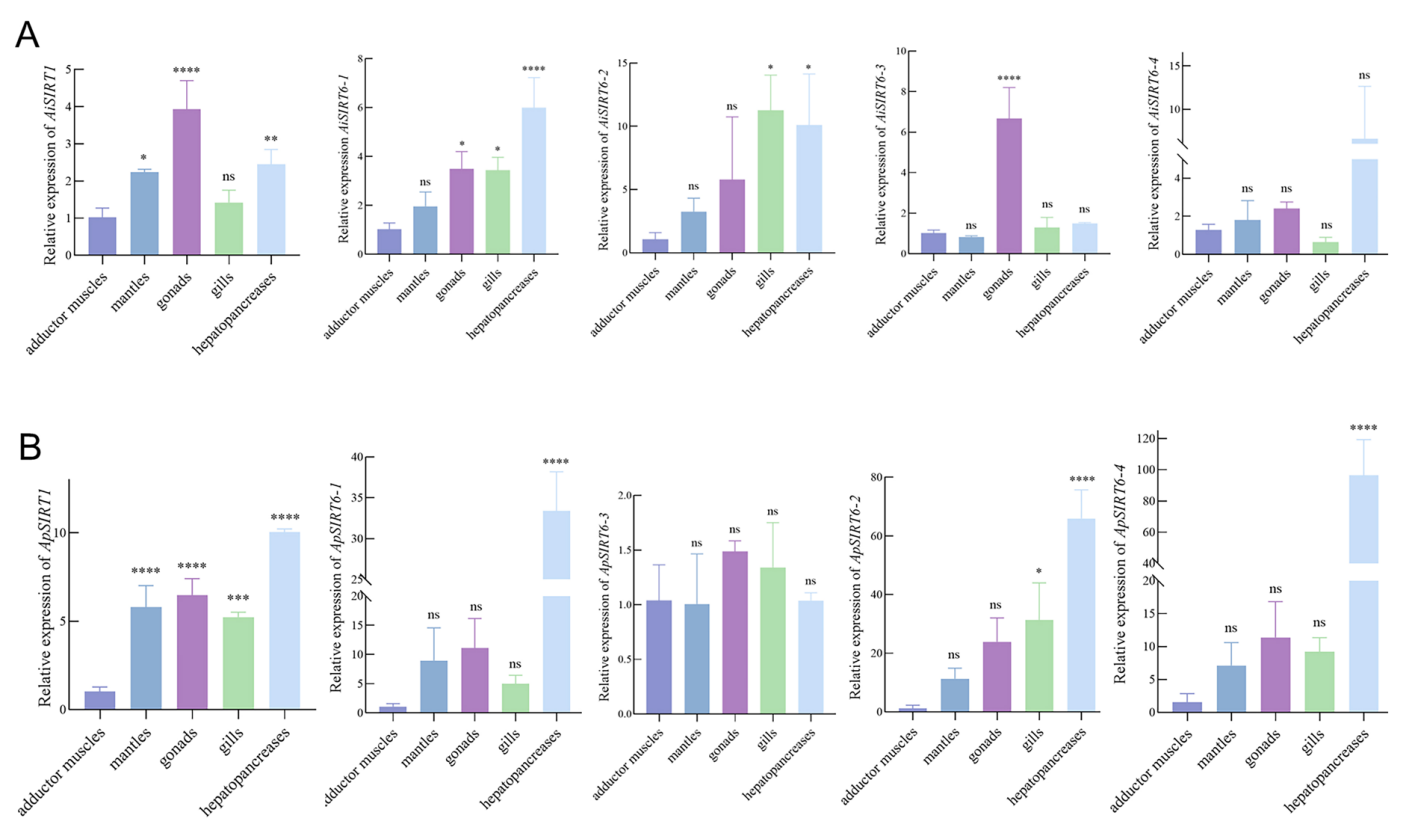
Tissue-specific expression of *SIRT1* and *SIRT6* genes in bay scallops **A** and in peruvian scallops **B** at 12 months. *P < 0.05; **P < 0.01; ***P < 0.001; ****P < 0.0001. Error bars represent standard deviations of the means of three replicates of each sample (*n* = 3)

The results revealed that the expression of *SIRT1* and *SIRT6* showed different expression patterns between the two scallops (Fig. 7). In the 12-month-old *A. irradians*, the expression of *AiSIRT1* was significantly higher expressed in the gonads, followed by the hepatopancreas and mantles (Fig. 7A); in *A. purpuratus*, the expression of *ApSIRT1* was the highest in the hepatopancreas, followed by the gonads and mantles (Fig. 7B). *AiSIRT6-1* was highly expressed in the hepatopancreas, followed by gonads and gills (Fig. 7A); the expression of *ApSIRT6-1* was highest in the hepatopancreas but showed no difference in the gonads and mantles (Fig. 7B). *AiSIRT6-2* was expressed predominantly in the gills and hepatopancreas, but the higher expression of *ApSIRT6-2* was significantly greater in the hepatopancreas (Fig. 7A), followed by the gills (Fig. 7B). *AiSIRT6-3* was expressed strongly in gonads, and its expression levels in other tissues were lower; *ApSIRT6-3* expression in all detected tissues did not reveal a significant change in peruvian scallops. *AiSIRT6-4* and *ApSIRT6-4* exhibited specific expression patterns in the hepatopancreas, and showed no difference in other tissues.

### Expression of the SIRT gene family and its related genes in the two scallops under NR

Transcriptome analysis revealed that *SIRT2* and *SIRT6* were significantly involved in nutrient stress response (*P* value < 0.05), followed by *SIRT1* and *SIRT3* (Table 3). The expression of *SIRT2* was significantly upregulated in the NR group of bay scallops, but it was slightly upregulated in peruvian scallops (*P* value < 0.05). The expression of *SIRT6* increased significantly in the NR group of peruvian scallops, and it was also upregulated in bay scallops but was less pronounced than that in peruvian scallops (*P* value < 0.05). The expression levels of the other three *SIRT* genes (*SIRT1*, *SIRT3* and *SIRT4*) showed little changes under NR stress in the two scallops. The expression of the *SIRT5* was enhanced only in peruvian scallops.

**Table 3:** Expression of the *SIRT* gene family under NR in two scallop species (*P* value < 0.05)

Gene	log_2_FC (Ai_E vs Ai_C)	log_2_FC (Ap_E vs Ap_C)
*SIRT1*	0.348	−0.494
*SIRT2*	9.284	1.264
*SIRT3*	0.117	−0.917
*SIRT4*	0.189	0.204
*SIRT5*	-0.710	1.349
*SIRT6*	1.091	4.220
*SIRT7*	0.563	0.860

Ai_C (Ap_C) represents the control group in bay scallops (peruvian scallops). Ai_E (Ap_E) represents NR group in bay scallops (peruvian scallops)

Next, the response of the two scallops to the *SIRT*-related genes in the NR was analyzed (*P* value < 0.05) (Table 4). *FoxO*, a downstream target gene of *SIRT1*, was upregulated in peruvian scallops under NR treatment. In addition, *BINP3*, *GADD45*, and *FASLG*, identified as downstream shotted genes of *FoxO*, exhibited different trends. *BINP3* and *GADD45* were both more robust in peruvian scallops. Conversely, *FASLG* was more down-regulated in bay scallops. Under NR, DNA replication, autophagy and apoptosis-related genes, which were affected by *SIRT1*, displayed dissimilar changes in the two scallop species. All DNA replication-related genes (*MCM2-8*, *PCNA*, and *POLA1*) were downregulated by NR conditions in both scallops, but peruvian scallops were even more down-regulated. Additionally, compared to those in bay scallops under NR, autophagy and antiapoptosis-related genes were significantly elevated in peruvian scallops. The expression of several genes responsible for insulin metabolism, AMPK and other stress metabolism pathways, such as *IGBP2*, *CREB3L4* and *HSP70*, was also obviously enhanced in the peruvian scallop. *PARP3*, considered antagonistic to *SIRT1*, was strongly downregulated in both scallops.

**Table 4:** *SIRT* -related DEGs response to NR in two scallop species (*P* value < 0.05)

Pathways	Genes	Description	log_2_FC (Ai_E vs Ai_C)	log_2_FC (Ap_E vs Ap_C)
FoxO signaling pathway	FoxO	forkhead box protein	–	1.625
	BINP3	BCL2/adenovirus E1B 19 kDa protein-interacting protein 3-like	–	1.492
	GADD45	growth arrest and DNA damage-inducible protein GADD45 beta-like	2.710	4.660
	FASLG	tumor necrosis factor ligand superfamily member 10-like	−2.444	−3.895
DNA replication pathway	MCM2	DNA replication licensing factor mcm 2	−0.668	−2.676
	MCM3	DNA replication licensing factor mcm 3	−0.883	−4.312
	MCM4	DNA replication licensing factor mcm 4	−1.012	−2.040
	MCM5	DNA replication licensing factor mcm 5	−1.219	−2.689
	MCM6	DNA replication licensing factor mcm 6	−0.510	−1.281
	MCM7	DNA replication licensing factor mcm 7	−0.385	−2.821
	MCM8	DNA replication licensing factor mcm 8	−0.948	-2.559
	POLA1	DNA polymerase alpha catalytic subunit-like	−1.406	−2.007
	PCNA	proliferating cell nuclear antigen-like	−0.750	−2.600
Autophagy signaling pathways	ATG10	autophagy-related protein 101-like	–	2.101
	ATG9A	autophagy-related protein 9A-like	–	2.519
	ULK2	serine/threonine-protein kinase unc-51-like	1.335	2.747
Apoptosis signaling pathway	BCL2L1	apoptosis regulator R1-like	2.301	3.6362
	BAX	Bcl-2 protein	1.157	−
Others	HSP70	stress-70 protein, mitochondrial-like	1.131	5.635
	CREB3L4	cyclic AMP-responsive element-binding protein 3-like protein 4	–	2.849
	UCP3	mitochondrial uncoupling protein 3-like	1.955	6.666
	PARP3	poly [ADP-ribose] polymerase 3-like	−1.211	−1.437
	insulin	insulin-like	−1.981	–
	IGFBP2	insulin-like growth factor-binding protein 5	–	3.407

Ai_C (Ap_C) represents the control group in bay scallops (peruvian scallops). Ai_E (Ap_E) represents NR group in bay scallops (peruvian scallops)

## Discussion

SIRT proteins are important functional proteins involved in aging processes, encompassing nutrient sensing, mitochondrial function, telomere maintenance and autophagy (Majmundar et al. 2010; Zhong et al. 2019). However, limited information is available concerning the role of the *SIRT* gene family in the aging function in marine animals. Peruvian scallops and bay scallops are taxonomically closely related bivalves with distinct life spans, offering a powerful opportunity for future functional studies on the modification of lifespan and the exploration of potential biomarkers for larger scallops. In the present study, the *SIRT* gene family was first used in a comprehensive genome-wide analysis in peruvian scallops and bay scallops, and molecular characteristics of *SIRT1* and *SIRT6* and their potential role on longevity were investigated in the two scallop species.

### Genome-wide identification provides insights into the evolution of the SIRT gene family

Comprehensive analyses of phylogeny, gene structure and motif composition helped to elucidate evolutionary relationships and genetic features of the *SIRT* gene family. In this study, different *SIRT* genes were identified between the two *Argopecten* scallops, with 15 in bay scallops but 11 in peruvian scallops. Consistently, the genes were classified into seven groups (*SIRT1-7*), which was similar to the findings of previous studies (Haigis and Yankner 2010; Wang et al. 2020). Gene characterization and structural differences between the two scallop species revealed that the genes were subjected to different selection pressures during evolution, resulting in chromosomal rearrangements and fusion events, thereby facilitating functional diversification of the *SIRT* gene family (Xu et al. 2012).

Gene duplication events are considered to be crucial for the evolution of gene families, as duplicate genes supply the essential components for the development of new gene functions, subsequently leading to the emergence of innovative roles (Moore and Purugganan 2003; Zhang et al. 2021a, b). More TD events were detected in bay scallops, signifying that TD primarily drove *SIRT* gene family expansion in bay scallops. *SIRTs* form a gene family of deacylases with a complex evolutionary history in eukaryotes, making it difficult to build a common *SIRT* repertoire. However, there are at least seven paralogs that may be dated to the last common ancestor of vertebrates (Opazo et al. 2023). The previous study confirmed this statement, increasing the number of *SIRT* family members from seven to ten in a Perciform fish, which served to disclose up to three gene copies of *SIRT3* (*SIRT3.1a*, *SIRT3.1b*, *SIRT3.2*) and two copies of *SIRT5* (*SIRT5a*, *SIRT5b*). *SIRT3.2* and *SIRT5b* enhanced expression on immune-relevant tissues and gills, indicating that they played a critical role in resisting environmental challenges. It was also discerned that *SIRT3.1a* and *SIRT3.1b* duplicates in two other percomorphaceae (*Parambassis ranga*, *Sphaeramia orbicularis*), which indicated that a gene retention might favor metabolic homeostasis (Simó et al. 2024). An extra copy of *SIR*2-promoting lifespan was also found in yeast and *C. elegans* (Tissenbaum and Guarenteet 2001). These results underscored the significance of gene duplication in driving both the expansion and reduction of gene families in vertebrates and invertebrates (Innan and Kondrashov 2010). The results of the selection test implemented on the *SIRT* genes showed that purifying selection occurred, suggesting that these genes underwent subfunctionalization or removed harmful functions, ultimately resulting in functional redundancy in peruvian scallops. For example, the expression of *ApSIRT6-3* was predominantly absent in tissues, potentially due to functional redundancy arising from purification processes. Further research will be focused on the characteristics of four copies of *SIRT6* in these two scallops in combination with functional experiments, reinforcing the importance of gene duplication shaping the landscape of the adaptable scallops.

### Sequence variations influence the activity of SIRT1 and SIRT6 in two scallop species

As positive longevity regulators, SIRT1 and SIRT6 may function to favor lifespan by promoting the deacetylation of histones and various transcription factors, including p53 and FoxO proteins. Previous studies have noted that synonymous and nonsynonymous substitutions can markedly change protein function through a wide range of mechanisms, such as protein level, translational accuracy, secretion efficiency, and posttranslational modifications (Walsh et al. 2020; Zhang et al. 2022). In bay scallops and peruvian scallops, the variations in sequences may also influence the deacetylation or phosphorylation modifications of *AiSIRT1* and *ApSIRT1*, which may play a potential role in the regulation of their activities through conformational changes (Cai et al. 2016; Nasrin et al. 2009). SIRT proteins shared a conserved central ‘SIR domain’, generally thought to be an enzymatic core. Specifically, the variations at K404 in the SIR domain of ApSIRT1 could change the activity of SIRT1, because lysine plays a central role in the process of deacetylation (Shahgaldi and Kahmini 2021). Also, some variations also occurred in SIRT6 in the two scallop species. In particular, the variations at H175 in AiSIRT6-4 in the SIR domain may change the activity of AiSIRT6-4 (Tennen et al. 2010). Disparities in the structures of the *SIRT1* and *SIRT6* genes could potentially underlie divergent functions within the biological processes in two scallop species longevity regulation. The nucleotide sequence data served as a foundation for comprehensive assessment and exploration of the fundamental functions of *SIRT1* and* SIRT6.*

The alignment results showed that SIRT1 and SIRT6 proteins conserved the necessary domains: GAGISTS (L/A) GIPDFR and YTQNID in the SIR domain. This was consistent with the findings of previous evolutionary studies, which showed that the *SIRT1* and *SIRT6* genes were highly conserved in bay scallops and peruvian scallops (Liszt et al. 2005). The N terminus of SIRT1 and SIRT6 played direct roles in the chemistry of deacetylation, for example by contributing to the binding of substrate or NAD^+^; such a role has been proposed for the N terminus of the *S. cerevisiae* sirtuin protein Hst2 (Min et al. 2001). Notably, the specific N terminus extension of AiSIRT1 and ApSIRT6-4 may play a role in different deacetylation activities in two scallop species. Phylogenetic tree revealed a close evolutionary relationship between *SIRT1* and *M. yessoensis* of *SIRT1*, whereas *SIRT6* genes from these two scallops formed a distinct branch separate from the *SIRT1* group genes. These findings suggested that *SIRT1* and *SIRT6* might exert distinct functions in certain pathways.

### Autoregulation conformation causes functional differences

On the basis of structural analysis, ApSIRT1 and AiSIRT1 were well-conserved in 3D structure. Previous study conducted that C-terminal and N-terminal extensions may autoregulate the NAD^+^ and acetyl-lysine substrate-binding sites, respectively (Zhao et al. 2003). Therefore, the differences in C-terminal and N-terminal extensions between ApSIRT1 and AiSIRT1 may cause dissociation or rearrangement of trimers, contributing to the different longevity functions or substrate specificities of ApSIRT1 and AiSIRT1. Alternatively, the NTE could contribute to SIRT6 function by promoting proper folding or protein stability (Tennen et al. 2010). However, the spatial structures of SIRT1 and SIRT6 in bay scallops and peruvian scallops were different, which may lead to the differences in the effects of longevity regulation.

Numerous studies have proposed that NLS and NES are positioned on the N-terminal extension of SIRT1 (Tanno et al. 2007). The unique N- and C-terminal regions are significant to mediate protein–protein interactions, subcellular localization and likely facilitate to SIRT-mediated biological processes (Zhang et al. 2021a, b). The N-terminal region may also interact with the active regulator of SIRT1 protein (Kim et al. 2007). Therefore, N-terminal difference, including the NLS sequence, between the two scallops may affect SIRT1 proteins activity to promote longevity in peruvian scallops. Integrating with functional analysis of mutants with progressive deletions from the N- and C-terminal and additional gene expression data is further required for a comprehensive understanding of the adaptive strategies of scallops in longevity regulation during the natural evolutionary process.

Different *SIRT6* isoforms represent a variant protein that differs in sequence from the constitutive protein, which would enlarge the diversity of *SIRT6* gene and adjust its subcellular localization and function. Although each isoform may have a distinct, probably slight, influence on cellular function, the combined impacts of the altered expression of all copies of *SIRT6* could have a deep effect on a series of physiological functions in various tissues and organs. There was no NLS on the C-terminal extensions of any of the four copies of SIRT6 in two scallop species. The validated subcellular location confirmed that SIRT1 and SIRT6 were both expressed in the cell nucleus, suggesting that the roles of SIRT1 and SIRT6 extended to the nucleus and interacted with related proteins. *FoxO* is phosphorylated by Akt and exported from the nucleus, resulting in its transcriptional repression (van der Horst et al. 2004). SIRT1 and SIRT6 have been shown to regulate the transcriptional activation of stress-resistance genes by FoxO proteins (Brunet et al. 2004; Chiang et al. 2012). Hence, the regulatory mechanism of the subcellular localization of SIRT1 and SIRT6 requires further study to help clarify the difference in longevity between these two scallops.

### The ApSIRT1 and ApSIRT6-2 might be anti-aging genes in peruvian scallops

At 12 months when bay scallops approached the senescence stage, the expression of *ApSIRT1* was increased significantly in the hepatopancreas, followed by gonad and mantle, which had more robust upregulation than *AiSIRT1*. In addition, *SIRT6-1*, *SIRT6-2*, *SIRT6-3,* and *SIRT6-4* were expressed in all examined tissues, indicating that they were not tissue-specific genes. However, the expression level of *SIRT6-2* was more powerful in both two scallops. Higher expression levels of *AiSIRT6-2* and *ApSIRT6-2* were found in hepatopancreas and gills. In bivalves, the hepatopancreas and gill are the predominant tissues in response to adverse environmental factors, so the higher expression of *SIRT6-2* in these tissues could be attributed to the breeding conditions. This indicates *SIRT6-2* may play a positive role in the protection of scallops against the adverse effects of environmental stress and function in various physiological processes in the two scallops (Cannuel et al. 2009; Sun et al. 2014). In old age, the function of the gonad for reproduction is gradually lost; similarly, the mantle gradually loses its age-related function for shell growth (Dell'Acqua et al. 2019; Wang et al. 2022). Therefore, the more significantly increased expression of *ApSIRT1* and *ApSIRT6-2* in these tissues of adults (at 12 months) could contribute to longevity promotion in peruvian scallops. Similarly, research by Wang et al. reported the higher expression of *ApFoxO*, which is regulated by *SIRT1* and *SIRT6*, in the gonad and mantle tissues of the peruvian scallops (Wang et al. 2022)*.* These results demonstrated that robust genes expression*,* such as *ApSIRT1* and *ApSIRT6-2* could play important roles in the lifespan of peruvian scallops.

### SIRT plays a role in longevity on anti-aging-related signaling pathways under NR

NR is a prospective and feasible strategy for the control of aging and aging-related diseases (Guarente 2013; Schenker et al. 2001). Our transcriptome analysis further revealed that two out of the seven *SIRT* genes were significantly upregulated under NR stress, indicating that *SIRT2* and *SIRT6* may be candidate genes in NR adaptation. However, the patterns of *SIRT2* and *SIRT6* upregulation differed between the two scallop species, which provided insights into the strategies for responding to nutrient stress in these invertebrates with distinct lifespans. Based on the above data, the different response to NR between bay scallops and peruvian scallops could result from their long-term adaptation to their environments with regard to food availability.

**Fig. 8:**
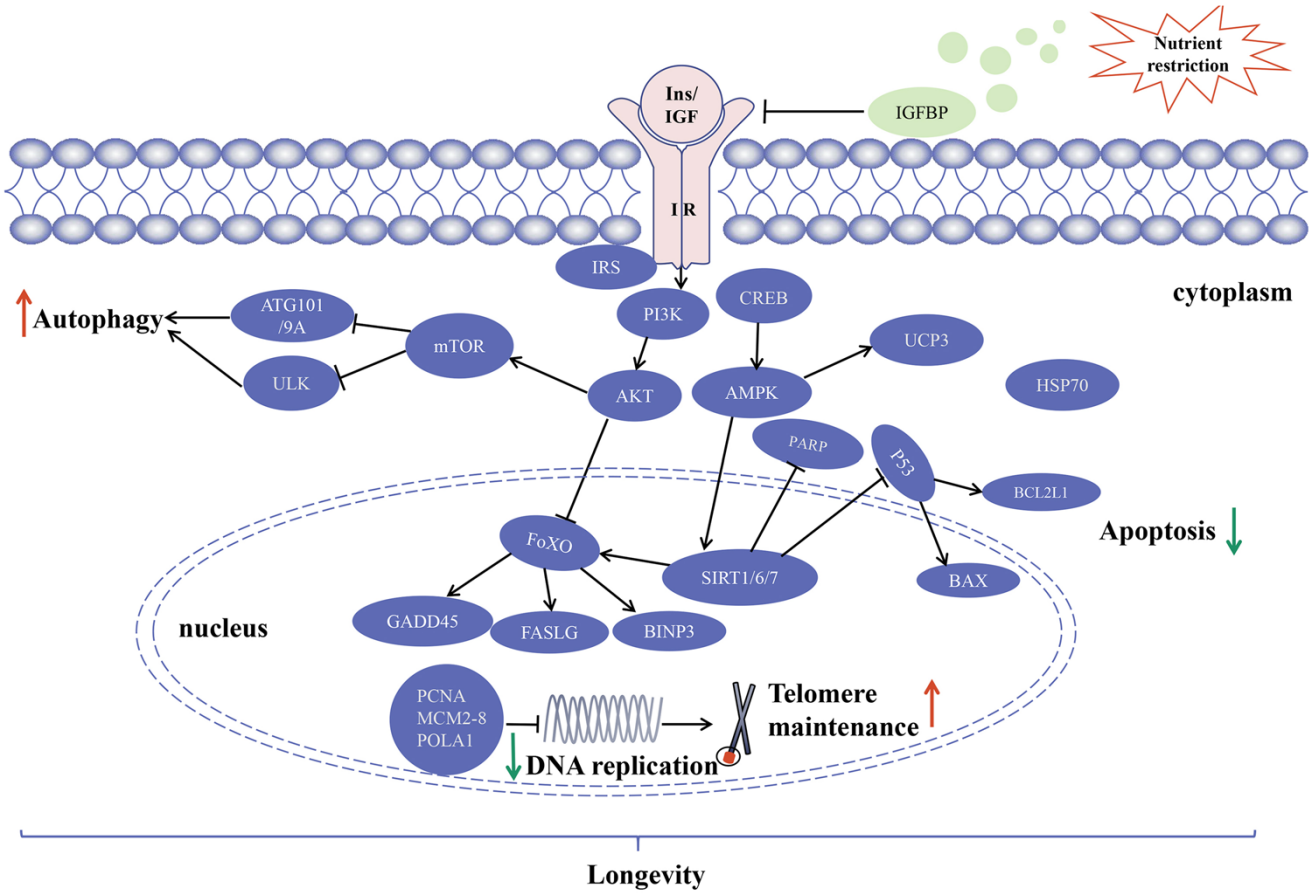
Potentially conserved regulatory network of the two scallop species. The black arrows represent the accelerating effect and the blunt arrows represent the inhibiting effect. The upward red arrows indicate the mechanism of promotion. The downward green arrows indicate the mechanism of inhibition

*SIRT* may delay cell senescence by inhibiting DNA damage, telomere shortening, apoptosis and insulin signaling pathway, and promoting autophagy and other mechanisms (Haigis and Yankner 2010; Wang et al 2020). The connections of cell nucleus *SIRT* and its anti-aging-related signaling pathways were first performed via transcriptome analysis to investigate the regulatory longevity mechanisms in these two scallops. Among the core genes regulated by *SIRT* genes*,* some anti-aging genes were promoted in the FoxO signaling pathway, autophagy signaling pathway, and apoptosis signaling pathway in peruvian scallops and bay scallops. This reveals the involvement of certain longevity genes in response to different environmental changes. When cells divide, telomere length shortens with each round of DNA replication (Han et al. 2016; Karlsen et al. 2022). Low levels of telomerase cause progressive telomere attrition, ultimately triggering DNA damage responses such as cell cycle arrest, apoptosis, impaired differentiation and/or senescence (Chakravarti et al. 2021). The expression of genes involved in DNA replication was significantly downregulated in the NR group, which indicated the NR group may have less DNA damage. All the upregulated or downregulated genes were highly robust in peruvian scallops, which may be one of the reasons for the longevity of these invertebrates. Consequently, we speculated that NR may prolong the lifespan of *Argopecten* scallops by decreasing DNA damage, upregulating *FoxO*-related genes and inducing the expression of autophagy-related and antiapoptotic genes (Fig. 8). Our results highlighted different and conserved nutrition-responsive genes in the two scallop species, which prompted us to characterize these longevity-promoting genes in subsequent research.

## Supplementary Information

Below is the link to the electronic supplementary material.

Supplementary file1 (PDF 2980 KB)

Supplementary file2 (DOCX 67 KB)

Supplementary file3 (XLSX 42 KB)

## Data Availability

Data will be made available on request.
