# Supplementary material for: Potential roles of the sirtuins in promoting longevity for larger Argopecten scallops
Source: Mar Life Sci Technol. 2025 Mar 4;7(2):284–301. doi: 10.1007/s42995-024-00269-3 (PMC12102419; doi:10.1007/s42995-024-00269-3)
Supplement: Supplementary file 1 — Supplementary file1 (PDF 2980 KB) [file 42995_2024_269_MOESM1_ESM.pdf]

Potential Roles of the Sirtuins in Promoting Longevity for Larger *Argopecten* Scallops

Yang Zhao[1,3], Junhao Ning[1], Yuan Wang[1,3], Guilong Liu[5], Xin Xu[5], Chunde Wang[1,4,*], Xia Lu[2,*]

[1]Research and Development Center for Efficient Utilization of Coastal Bioresources, Yantai Institute of Coastal Zone Research, Chinese Academy of Sciences, Yantai 264003, China

[2] School of Ocean, Yantai University, Yantai 264005, China

[3]University of Chinese Academy of Sciences, Beijing 100049, China

[4]College of Marine Science and Engineering, Qingdao Agricultural University, Qingdao 266109, China

[5]Yantai Spring-Sea AquaSeed, Ltd., Yantai 264006, China

[*] Correspondence：

Dr. Chunde Wang, chundewang2007@163.com

Dr. Xia Lu, luxia_0202@163.com

Supplemental Information

Supplemental Figs. S1-S13

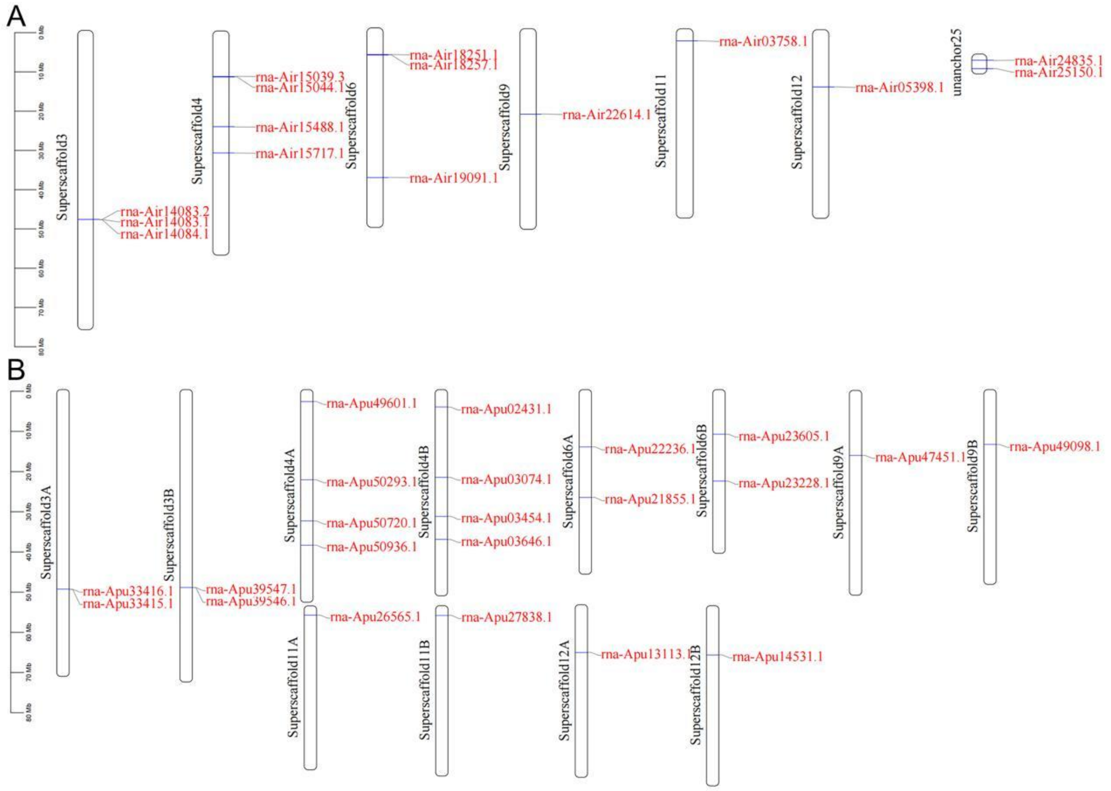


**Fig. S1.** Chromosome distribution of *SIRT* genes in the two scallop species. (A) the bay scallop; (B) the Peruvian scallop with two homologous chromosomes.

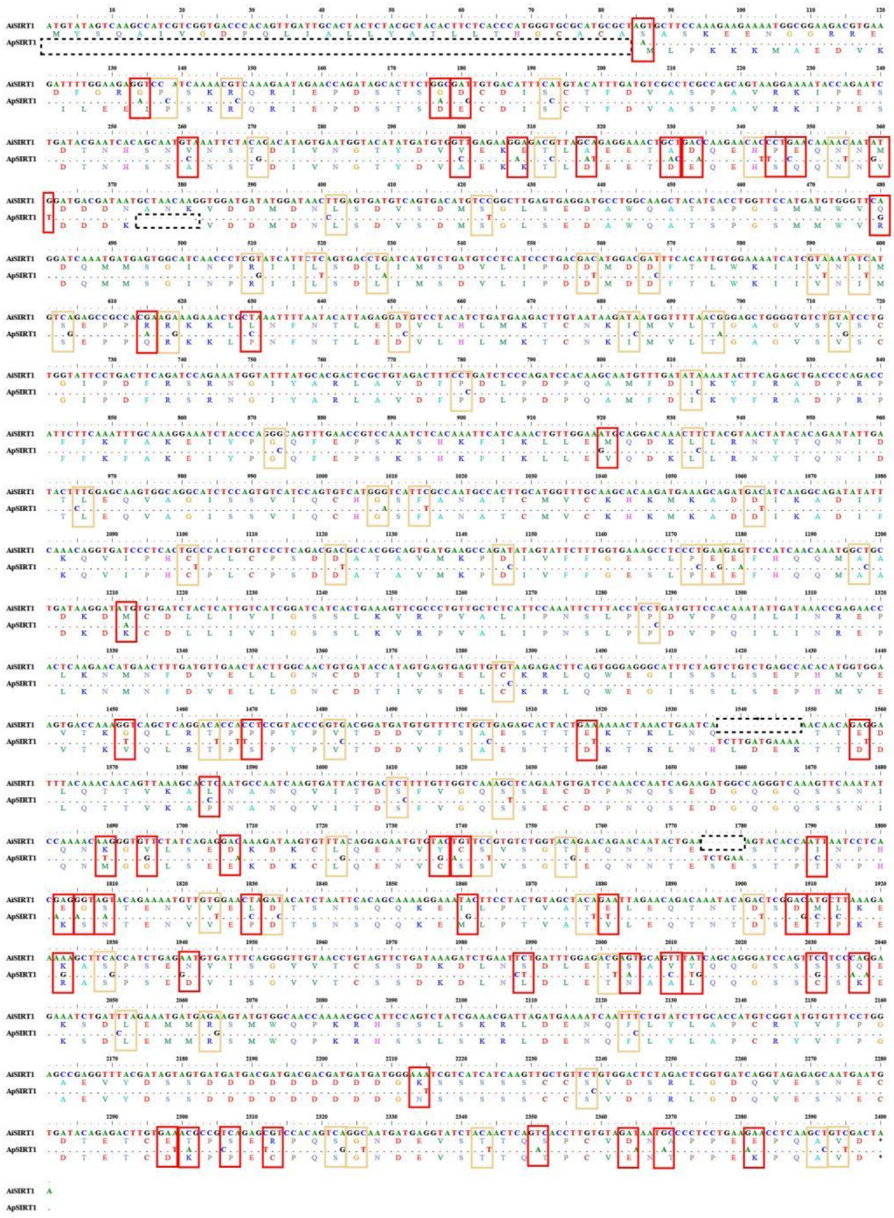

**Fig. S2.** Nucleotide and amino acid variations between *AiSIRT1* and *ApSIRT1*. The red box and orange box indicate the nonsynonymous and synonymous variations. The dash box indicates Indels.

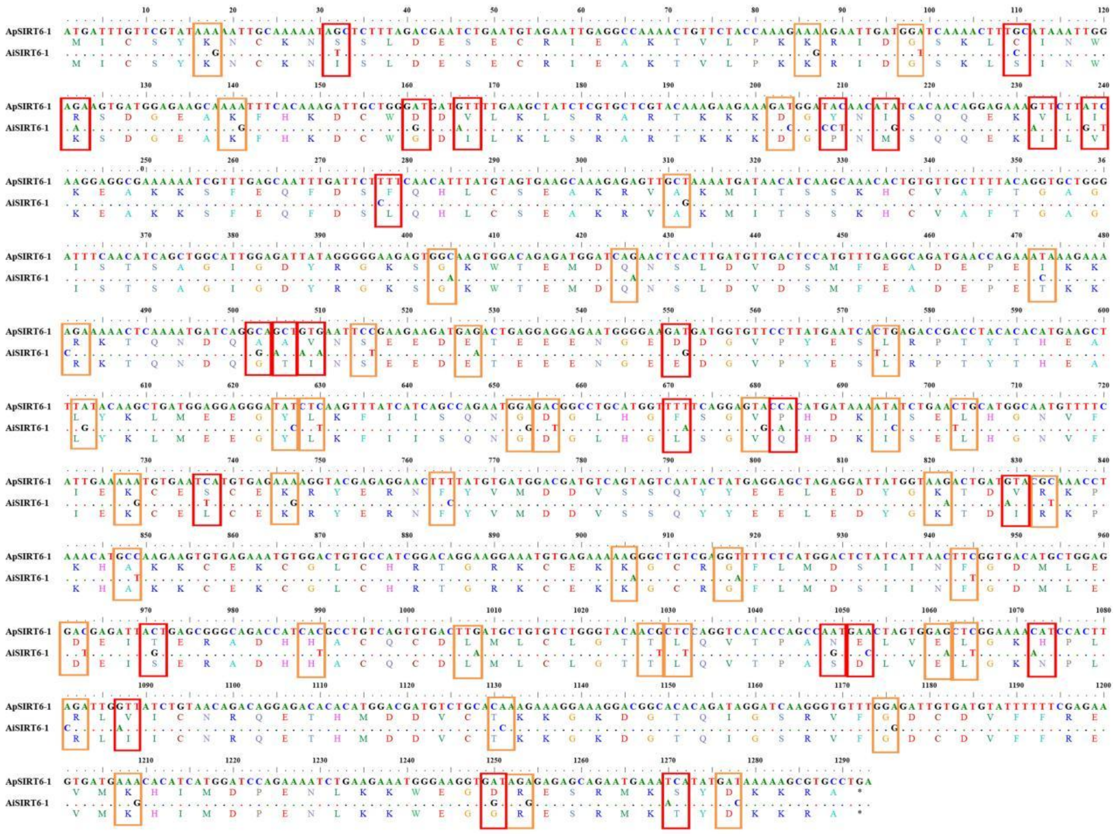

**Fig. S3.** Nucleotide and amino acid variations between *AiSIRT6-1* and *ApSIRT6-1*. The red box and orange box indicate the nonsynonymous and synonymous variations. The dash box indicates Indels.

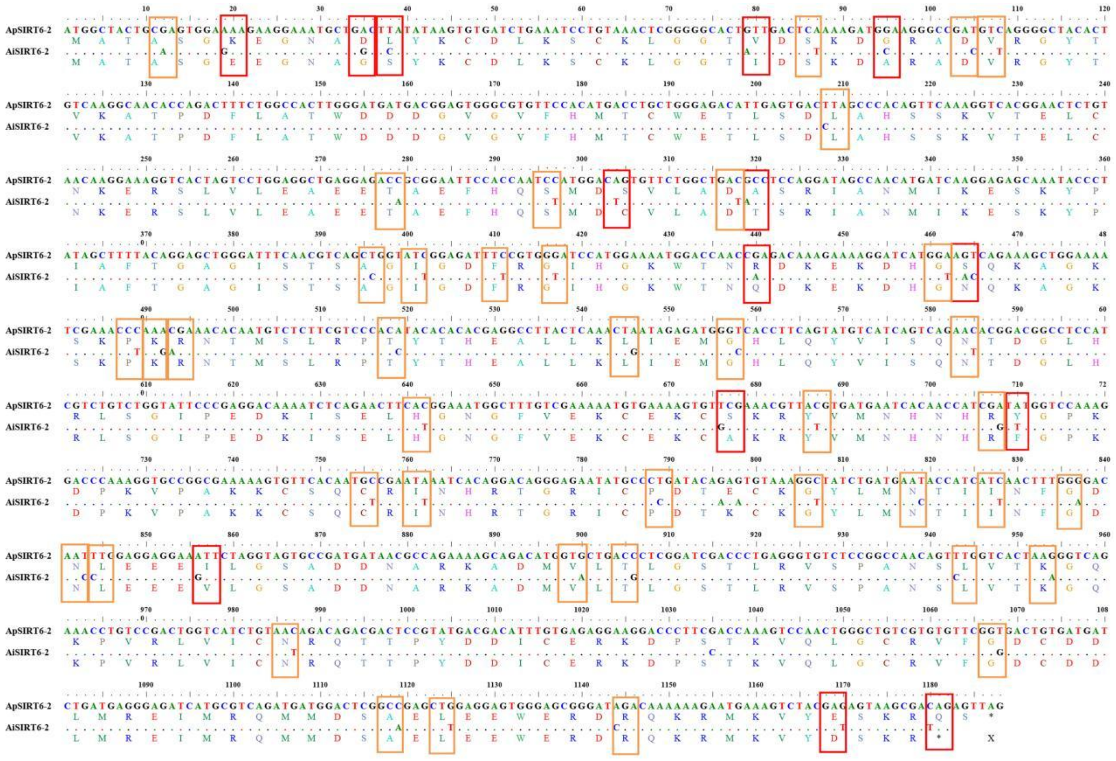

**Fig. S4.** Nucleotide and amino acid variations between *AiSIRT6-2* and *ApSIRT6-2*. The red box and orange box indicate the nonsynonymous and synonymous variations.

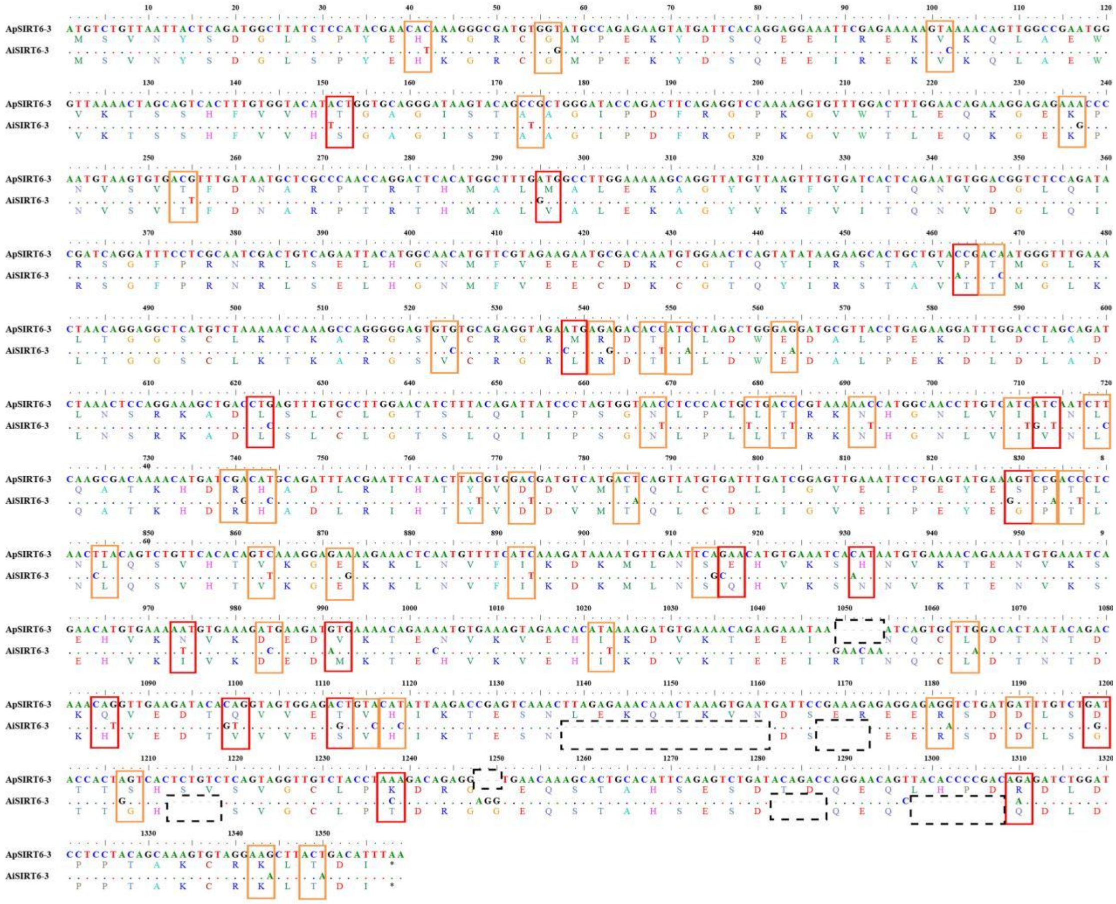

**Fig. S5.** Nucleotide and amino acid variations between *AiSIRT6-3* and *ApSIRT6-3*. The red box and orange box indicate the nonsynonymous and synonymous variations. The dash box indicates Indels.

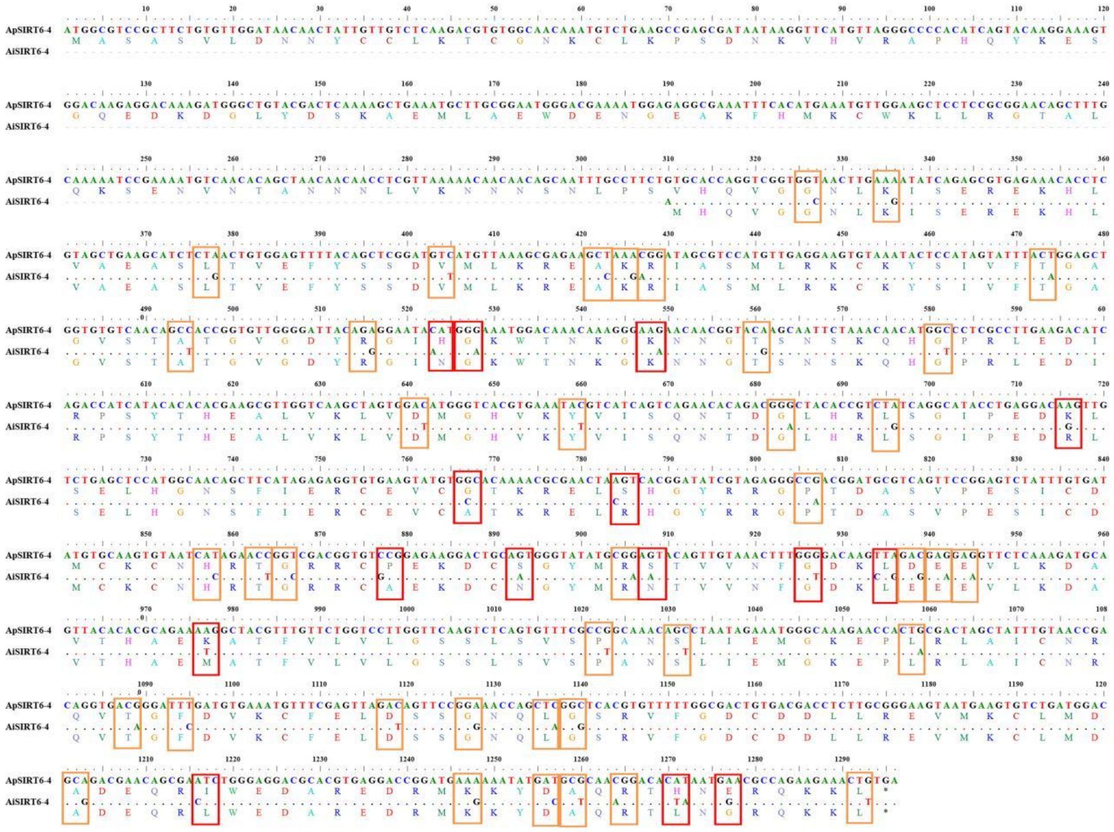

**Fig. S6.** Nucleotide and amino acid variations between *AiSIRT6-4* and *ApSIRT6-4*. The red box and orange box indicate the nonsynonymous and synonymous variations. The dash box indicates Indels.

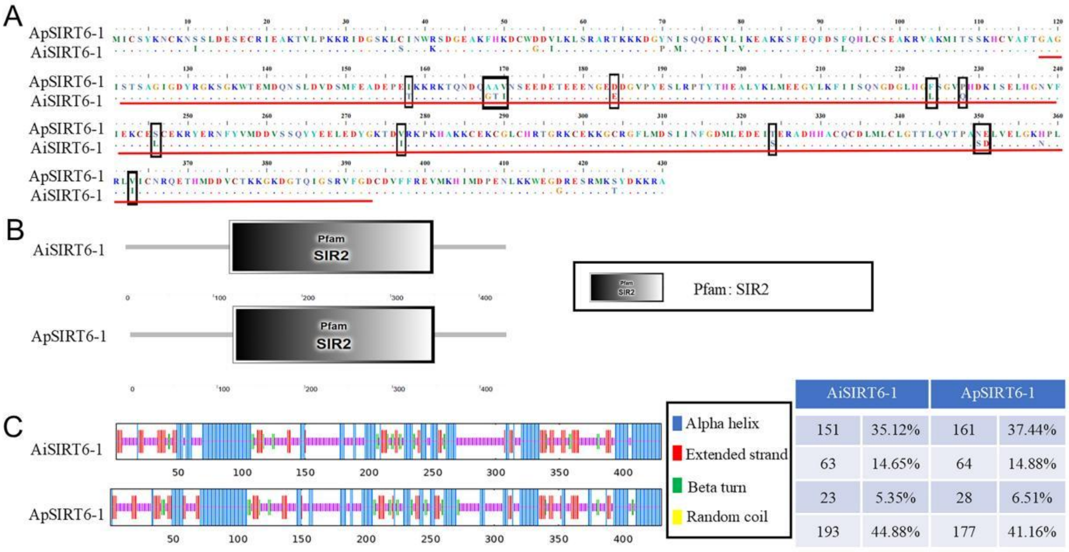


**Fig. S7.** The sequence alignments (A), the signal peptide (B), the structure domains (C) the secondary structure (D) of AiSIRT6-1 and ApSIRT6-1. The changes in the SIRT domain and amino acids changes are marked by red line and black box, respectively.

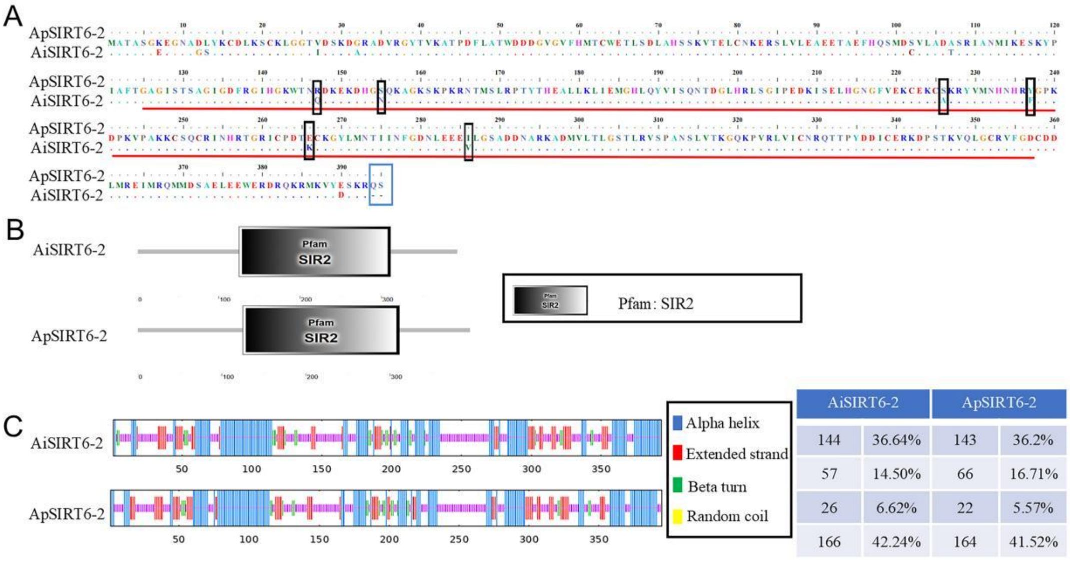


**Fig. S8.** The sequence alignments (A), the signal peptide (B), the structure domains (C) the secondary structure (D) of AiSIRT6-2 and ApSIRT6-2. The SIRT domain and the amino acids changes are marked by red line and black box, respectively. The blue box represents the InDels.

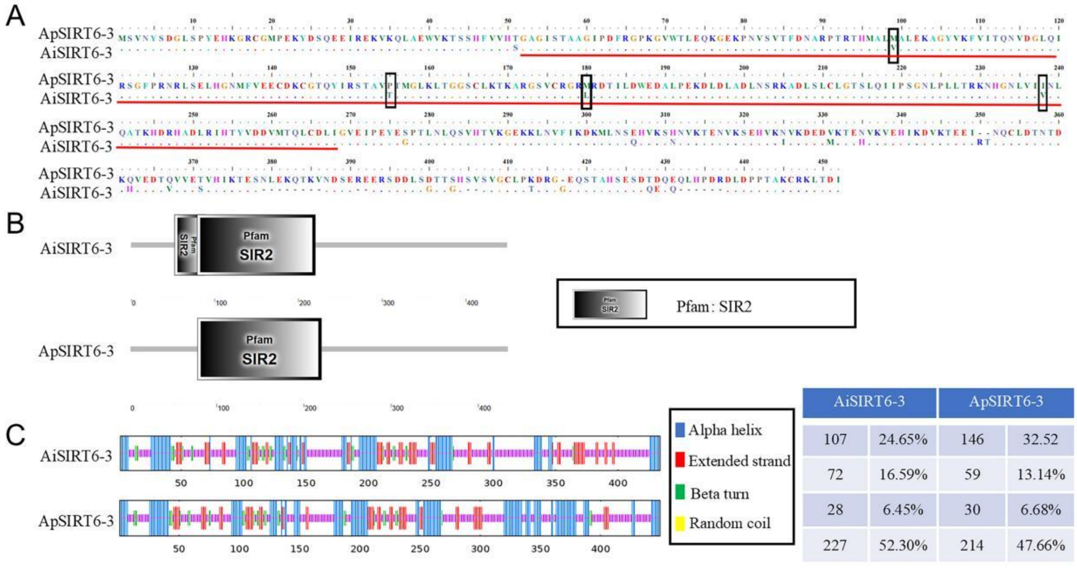


**Fig. S9.** The sequence alignments (A), the signal peptide (B), the structure domains (C) the secondary structure (D) of AiSIRT6-3 and ApSIRT6-3. The SIRT domain and the amino acids changes are marked by red line and black box, respectively.

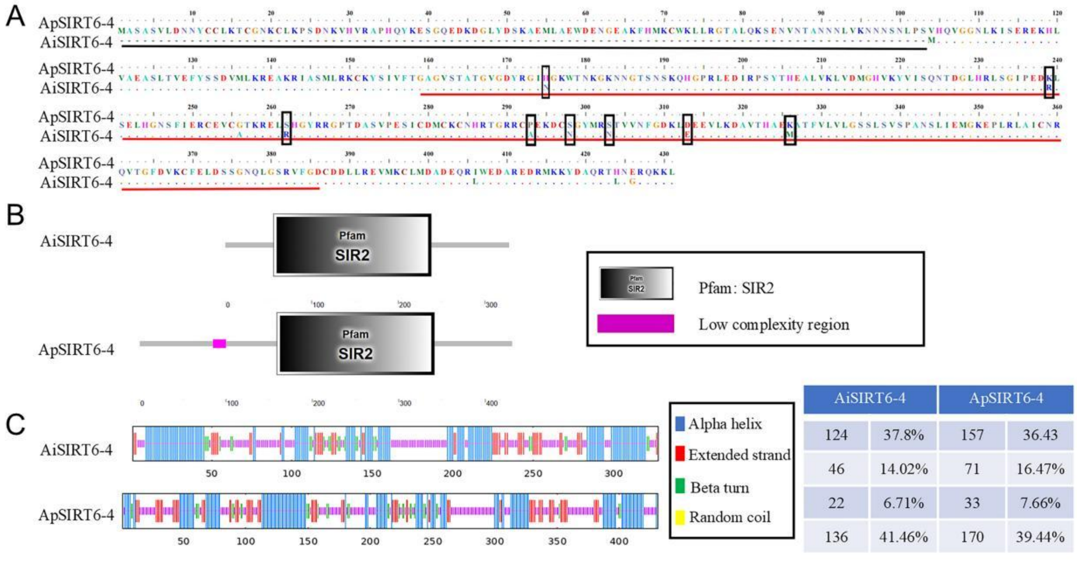


**Fig. S10.** The sequence alignments (A), the signal peptide (B), the structure domains (C) the secondary structure (D) of AiSIRT6-4 and ApSIRT6-4. The SIRT domain and the amino acids changes are marked by red line and black box, respectively. The black line represents the InDels.

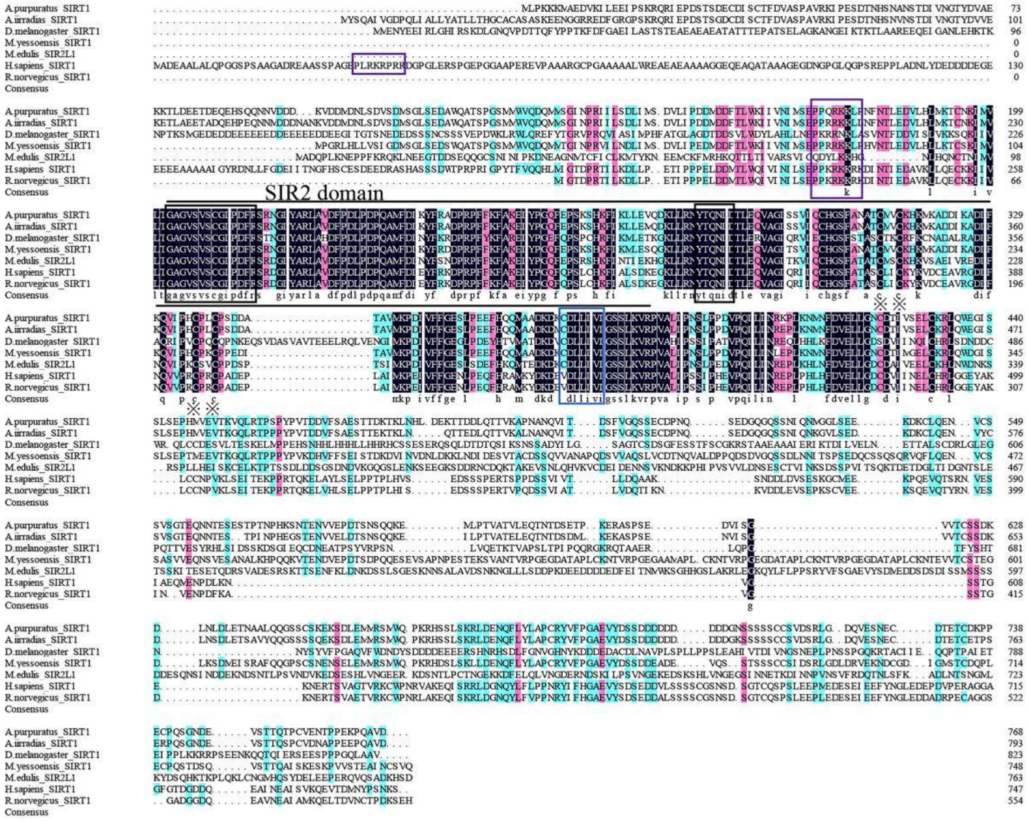

**Fig. S11.** Amino acid sequence alignment of SIRT1 proteins in two scallop species. The predicted SIR2 domain was lined in black. The Zn binding sites were indicated by the “※”. The blue box indicated the NES. The purple box indicated the NLS. The black box was the necessary motif.

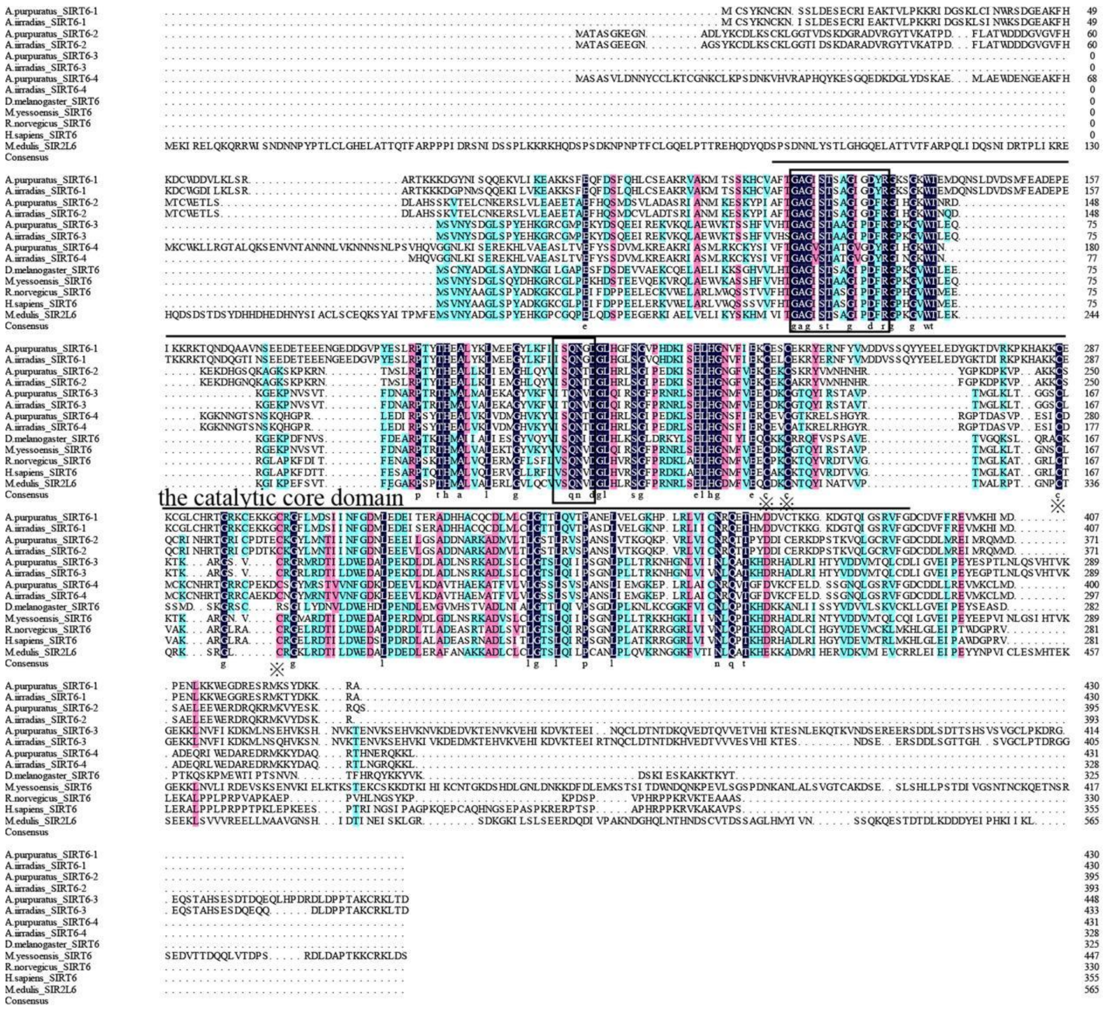


**Fig. S12.** Amino acid sequence alignment of SIRT6 proteins in two scallop species. The predicted SIR2 domain was lined in black. The Zn binding sites were indicated by the "※". The blue box indicated the NLS. The black box was the necessary motif.

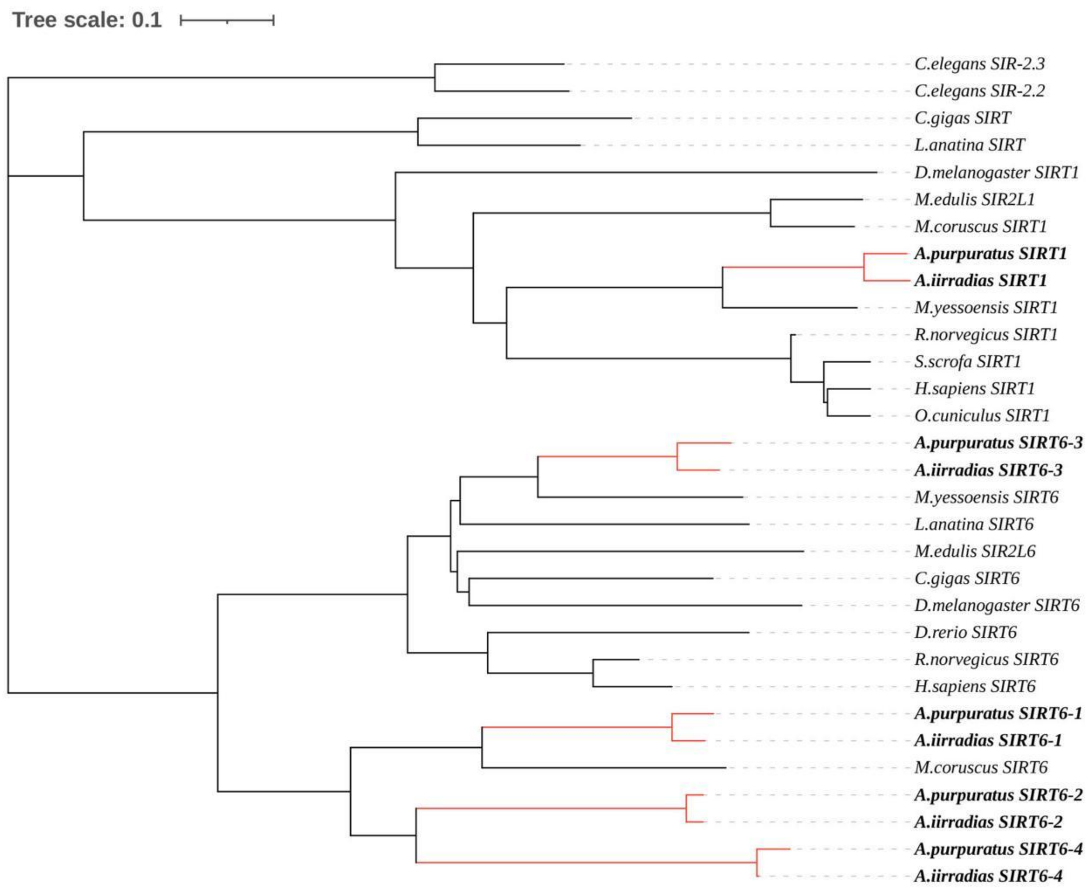


**Fig. S13.** The phylogenetic analysis for *SIRT1* and *SIRT6* in two scallop species.
