# Supplementary material for: Potential roles of the sirtuins in promoting longevity for larger Argopecten scallops
Source: Mar Life Sci Technol. 2025 Mar 4;7(2):284–301. doi: 10.1007/s42995-024-00269-3 (PMC12102419; doi:10.1007/s42995-024-00269-3)
Supplement: Supplementary file 2 — Supplementary file2 (DOCX 67 KB) [file 42995_2024_269_MOESM2_ESM.docx]

Potential Roles of the Sirtuins in Promoting Longevity for Larger *Argopecten* Scallops

Yang Zhao^1,3^, Junhao Ning^1^, Yuan Wang^1,3^, Guilong Liu^5^, Xin Xu^5^, Chunde Wang^1, 4,*^, Xia Lu^2,*^

^1^Research and Development Center for Efficient Utilization of Coastal Bioresources, Yantai Institute of Coastal Zone Research, Chinese Academy of Sciences, Yantai 264003, China

^2^ School of Ocean, Yantai University, Yantai 264005, China

^3^ University of Chinese Academy of Sciences, Beijing 100049, China

^4^College of Marine Science and Engineering, Qingdao Agricultural University, Qingdao 266109, China

^5^Yantai Spring-Sea AquaSeed, Ltd., Yantai 264006, China

^*^ Correspondence：

Dr. Chunde Wang, chundewang2007@163.com

Dr. Xia Lu, luxia_0202@163.com

Supplemental Information

Supplemental Tables S1-S5

**Table S1** Primers used in the present study

| Primer name | Sequence (5’ -3’) | Purpose |
| --- | --- | --- |
| ApSIRT1-F | ATGCTTCCAAAGAAGAAAATGGCG | ORFamplification |
| ApSIRT1-R | TTAGTCAACGGCTTGAGGTTCT | ORF amplification |
| AiSIRT1-F | ATGTATAGTCAAGCCATCGTCG | ORF amplification |
| AiSIRT1-R | TTAGTCGACAGCTTGAGGTTCT | ORF amplification |
| ApSIRT6-1-F | ATGATTTGTTCGTATAAAAATTGCAAAA | ORF amplification |
| ApSIRT6-1-R | TCAGGCACGCTTTTTATCATATGAT | ORF amplification |
| AiSIRT6-1-F | ATGATTTGTTCGTATAAGAATTGCAAA | ORF amplification |
| AiSIRT6-1-R | TCAGGCACGCTTTTTGTCATATG | ORF amplification |
| ApSIRT6-2-F | ATGGCTACTGCGAGTGGAAAAG | ORF amplification |
| ApSIRT6-2-R | CTAACTCTGTCGCTTACTCTCG | ORF amplification |
| AiSIRT6-2-F | ATGGCTACTGCAAGTGGAGAAG | ORF amplification |
| AiSIRT6-2-R | CTATCGCTTACTATCGTAGACTTTC | ORF amplification |
| ApSIRT6-3-F | ATGTCTGTTAATTACTCAGATGGCTT | ORF amplification |
| ApSIRT6-3-R | TTAAATGTCAGTAAGCTTCCTACAC | ORF amplification |
| AiSIRT6-3-F | ATGTCTGTTAATTACTCAGATGGCTT | ORF amplification |
| AiSIRT6-3-R | TTAAATGTCTGTAAGTTTCCTACACTT | ORF amplification |
| ApSIRT6-4-F | ATGGCGTCCGCTTCTGTGTTG | ORF amplification |
| ApSIRT6-4-R | TCACAGTTTCTTCTGGCGTTCA | ORF amplification |
| AiSIRT6-4-F | ATGTCAGCACAGCTAACAACAAC | ORF amplification |
| AiSIRT6-4-R | TCAAAGTTTCTTCTGGCGTCCA | ORF amplification |
| ApSIRT1-F | TGTCTGGCTTGAGTGAGGATGC | RT-qPCR |
| qApSIRT1-R | GATCCGAGGGTTGATGCCACTC | RT-qPCR |
| qApSIRT6-1-F | CTTCGGTGACATGCTGGAGGAC | RT-qPCR |
| qApSIRT6-1-R | GTGACCTGGAGCGTTGTACCC | RT-qPCR |
| qApSIRT6-2-F | TGTCGTGTGTTCGGTGACTGTG | RT-qPCR |
| qApSIRT6-2-R | TGTCTATCCCGCTCCCACTCC | RT-qPCR |
| qApSIRT6-3-F | AGCACTGCTGTACCGACAATGG | RT-qPCR |
| qApSIRT6-3-R | CAGGTAACGCATCCTCCCAGTC | RT-qPCR |
| qApSIRT6-4-F | TGCGGAATGGGACGAAAATG | RT-qPCR |
| qApSIRT6-4-R | GTGCACAGAAGGCAAATTGC | RT-qPCR |
| qAiSIRT1-F | ATGACGATGACGACGATGATGATGG | RT-qPCR |
| qAiSIRT1-R | TGCCTGACTGTGGACGCTCTG | RT-qPCR |
| qAiSIRT6-1-F | GAGATTAGTGAGCGGGCAGACC | RT-qPCR |
| qAiSIRT6-1-R | CTGGCTGGTGTGACCTGAAGAG | RT-qPCR |
| qAiSIRT6-2-F | ACCAAAGTCCAACTGGGCTGTC | RT-qPCR |
| qAiSIRT6-2-R | GCTCTGCCGAGTCCATCATCTG | RT-qPCR |
| qAiSIRT6-3-F | TGCGTGCCGATCATGTTTTGTC | RT-qPCR |
| qAiSIRT6-3-R | AGCACTGCTGTACCGACAATGG | RT-qPCR |
| qAiSIRT6-4-F | ACATGGTCCTCGCCTTGAAGAC | RT-qPCR |
| qAiSIRT6-4-R | GCCTGACAGACGGTGTAGTCC | RT-qPCR |
| EF-α-F | CCTTTGCCCCATCTCAACTGTC | RT-qPCR |
| EF-α-R | CAGCAACGTTTCCTCTCTTCAATTC | RT-qPCR |

**Table S2**

Physicochemical properties of the identified *SIRT* genes in two scallop species

| Gene symbol | Superscaffold | CDS  size  (bp) | Number of amino acids  (aa) | Molecular weight  (kDa) | PI | GRAVY |
| --- | --- | --- | --- | --- | --- | --- |
| rna-Air15044.1 | Superscaffold4 | 2382 | 793 | 87.62534 | 4.36 | -0.558 |
| rna-Apu50293.1 | Superscaffold9A | 2307 | 768 | 85.08454 | 4.36 | -0.627 |
| **rna-Apu03074.1** | Superscaffold10B | 2307 | 768 | 85.08454 | 4.36 | -0.627 |
| rna-Air14084.1 | Superscaffold3 | 1056 | 351 | 39.34416 | 5.97 | -0.367 |
| rna-Air14083.2 | Superscaffold3 | 1311 | 436 | 48.98381 | 5.83 | -0.616 |
| rna-Air14083.1 | Superscaffold3 | 1362 | 453 | 50.94498 | 5.89 | -0.632 |
| **rna-Apu39546.1** | Superscaffold4B | 1233 | 410 | 46.23539 | 7.92 | -0.319 |
| rna-Apu33415.1 | Superscaffold3A | 1392 | 463 | 50.91922 | 5.61 | -0.284 |
| rna-Apu39547.1 | Superscaffold4A | 1326 | 441 | 49.55257 | 5.93 | -0.62 |
| **rna-Apu33416.1** | Superscaffold3B | 1326 | 441 | 49.50757 | 6.14 | -0.631 |
| rna-Air19091.1 | Superscaffold6 | 2031 | 676 | 74.67022 | 9.94 | -0.777 |
| rna-Apu21855.1 | Superscaffold21A | 2238 | 745 | 81.93556 | 9.84 | -0.719 |
| **rna-Apu23228.1** | Superscaffold22B | 2148 | 715 | 78.70569 | 9.9 | -0.748 |
| rna-Air24835.1 | unanchor25 | 867 | 288 | 30.5701 | 7.68 | -0.043 |
| rna-Air05398.1 | Superscaffold12 | 933 | 310 | 34.68305 | 9.41 | -0.238 |
| rna-Apu13113.1 | Superscaffold17A | 933 | 310 | 34.94630 | 9.27 | -0.267 |
| **rna-Apu14531.1** | Superscaffold18B | 921 | 306 | 34.44086 | 9.35 | -0.22 |
| rna-Air18251.1 | Superscaffold6 | 966 | 321 | 35.28841 | 8.34 | -0.292 |
| rna-Air25150.1 | unanchor25 | 798 | 265 | 28.36518 | 6.14 | -0.154 |
| rna-Air18257.1 | Superscaffold6 | 966 | 321 | 35.32037 | 7.62 | -0.296 |
| rna-Apu22236.1 | Superscaffold21A | 909 | 302 | 32.96555 | 6.38 | -0.279 |
| **rna-Apu23605.1** | Superscaffold22B | 966 | 321 | 35.26134 | 8.06 | -0.3 |
| rna-Air03758.1 | Superscaffold11 | 1305 | 434 | 48.33141 | 6.07 | -0.642 |
| rna-Air15039.3 | Superscaffold4 | 1038 | 345 | 38.49799 | 8.76 | -0.495 |
| rna-Air15717.1 | Superscaffold4 | 1182 | 393 | 43.98094 | 8.09 | -0.676 |
| rna-Air22614.1 | Superscaffold9 | 1293 | 430 | 49.07559 | 6.11 | -0.821 |
| **rna-Apu02431.1** | Superscaffold10B | 1296 | 431 | 48.35283 | 8.62 | -0.694 |
| rna-Apu49601.1 | Superscaffold9A | 1284 | 427 | 48.16854 | 7.25 | -0.646 |
| **rna-Apu27838.1** | Superscaffold26B | 1305 | 449 | 50.30455 | 5.93 | -0.715 |
| rna-Apu26565.1 | Superscaffold25A | 1311 | 436 | 49.07622 | 5.86 | -0.711 |
| rna-Apu47451.1 | Superscaffold7A | 876 | 291 | 33.63872 | 5.11 | -0.913 |
| **rnaApu-49098.1** | Superscaffold8B | 1293 | 430 | 49.32668 | 6.03 | -0.858 |
| **rna-Apu03454.1** | Superscaffold10B | 1188 | 395 | 44.31323 | 8.11 | -0.713 |
| rna-Apu50720.1 | Superscaffold9A | 1188 | 395 | 44.29723 | 8.11 | -0.706 |
| rna-Air15488.1 | Superscaffold4 | 2190 | 729 | 81.49748 | 9.24 | -0.663 |
| **rna-Apu03646.1** | Superscaffold10B | 2196 | 731 | 81.33716 | 9.24 | -0.656 |
| rna-Apu50936.1 | Superscaffold9A | 2196 | 731 | 81.33622 | 9.29 | -0.657 |

Notice: *ApSIRT* within a box are two duplicated. The bold and underlined ones come from the homologous chromosomes.

**Table S3**

Ks, Ka, and Ka/Ks values calculated for paralogous SIRT1 and SIRT6 gene pairs in two scallop species

| Genes | | Ka | Ks | Ka/Ks |
| --- | --- | --- | --- | --- |
| AiSIRT1 | ApSIRT1 | 0.047151524 | 0.164503076 | 0.286630046 |
| ApSIRT6-1 | AiSIRT6-1 | 0.022253824 | 0.152615699 | 0.145816087 |
| ApSIRT6-2 | AiSIRT6-2 | 0.02944245 | 0.177975337 | 0.165429943 |
| ApSIRT6-3 | AiSIRT6-3 | 0.01437567 | 0.18206867 | 0.078957409 |
| ApSIRT6-4 | AiSIRT6-4 | 0.054331272 | 0.264315819 | 0.205554372 |

**Table S4**

Online prediction of subcellular localization of SIRT1 and SIRT6 proteins

| Genes | Cell-PLoc 2.0 | WoLF PSORT | Ks | Ka/Ks |
| --- | --- | --- | --- | --- |
| AiSIRT1 | Cytoplasm, Nucleus | nucl: 8.5, cyto_nucl: 8.5, extr_plas: 8.5, extr: 8, cyto: 7.5, plas: 7, E.R.: 1 | | |
| ApSIRT1 | Nucleus. | nucl: 19.5, cyto_nucl: 15.5, cyto: 8.5, plas: 4 | | |
| AiSIRT6-1 | Nucleus | cyto: 19, nucl: 11, plas: 2 | | |
| AiSIRT6-2 | Cytoplasm | cyto: 20.5, cyto_nucl: 13.5, nucl: 5.5, mito: 4, extr: 1, lyso: 1 | | |
| AiSIRT6-3 | Nucleus | cyto: 21.5, cyto_nucl: 15.3333, cyto_mito: 12.6667, nucl: 7, mito: 2.5, pero: 1 | | |
| AiSIRT6-4 | Nucleus | extr: 13, mito: 11, E.R._mito: 7, cyto: 4, nucl: 2, lyso: 1 | | |
| ApSIRT6-1 | Nucleus | nucl: 16, cyto: 13, plas: 2, extr: 1 | | |
| ApSIRT6-2 | Cytoplasm,Nucleus | cyto: 19, mito: 6, nucl: 4, plas: 1, extr: 1, lyso: 1 | | |
| ApSIRT6-3 | Nucleus | cyto: 19.5, cyto_nucl: 13.5, nucl: 6.5, mito: 5, pero: 1 | | |
| ApSIRT6-4 | Nucleus | cyto: 12, cyto_nucl: 11.5, mito: 8, nucl: 7, extr: 2, golg: 2, lyso: 1 | | |
